# Comparative efficacy of different renin angiotensin system blockade therapies in patients with IgA nephropathy: a Bayesian network meta-analysis of 17 RCTs

**DOI:** 10.7717/peerj.11661

**Published:** 2021-07-06

**Authors:** Zhihao Huo, Huizhen Ye, Peiyi Ye, Guanqing Xiao, Zhe Zhang, Yaozhong Kong

**Affiliations:** 1 Nephrology Department, The First People’s Hospital of Foshan, Foshan, China; 2 National Clinical Research Center for Kidney Disease, State Key Laboratory of Organ Failure Research, Guangdong Provincial Clinical Research Center for Kidney Disease, Renal Division, Nanfang Hospital, Southern Medical University, Guangzhou, China; 3 Staff Health Care Department, Sun Yat-sen University Cancer Center, State Key Laboratory of Oncology in South China, Collaborative Innovation Center for Cancer Medicine, Guangzhou, China

**Keywords:** Bayesian network analysis, IgA nephropathy, Proteinuria, Renoprotective effect, ACEI/ARB

## Abstract

**Background:**

IgA nephropathy (IgAN) is still one of the most prevalent forms of primary glomerulonephritis globally. However, no guidelines have clearly indicated which kinds of renin angiotensin system blockade therapies (ACEIs or ARBs or their combination) in patients with IgAN result in a greater reduction in proteinuria and a better preservation of kidney function. Thus, we conducted a Bayesian network analysis to evaluate the relative effects of these three therapy regimens in patients with IgAN.

**Methods:**

The protocol was registered in PROSPERO with ID CRD42017073726. We comprehensively searched the PubMed, the Cochrane Library, Embase, China Biology Medicine disc, WanFang and CNKI databases for studies published since 1993 as well as some grey literature according to PICOS strategies. Pairwise meta-analysis and Bayesian network analysis were conducted to evaluate the effect of different regimens.

**Results:**

Seventeen randomized controlled trials (RCTs) involving 1,006 patients were analyzed. Co-administration of ACEIs and ARBs had the highest probability (92%) of being the most effective therapy for reducing proteinuria and blood pressure, but ACEIs would be the most appropriate choice for protecting kidney function in IgAN.

**Conclusion:**

The combination of ACEIs and ARBs seems to have a significantly better antiproteinuric effect and a greater reduction of blood pressure than ACEI or ARB monotherapy in IgAN. ACEIs appear to be a more renoprotective therapy regimen among three therapies.

## Introduction

IgA nephropathy (IgAN) is the most prevalent form of primary glomerulonephritis globally, and remains a leading cause of chronic kidney disease (CKD) and kidney failure (Lai et al., 2016; Rodrigues, Haas & Reich, 2017). Among patients with IgAN, approximately 30%–50% deteriorate to end-stage renal disease (ESRD) within 20 to 30 years due to glomerulosclerosis, podocyte injury and tubulointerstitial fibrosis (Lai et al., 2016; Maixnerova & Tesar, 2020; Moriyama et al., 2014), and 1.2% of IgAN rapidly deteriorate in kidney function, resulting in acute kidney injury (Kveder et al., 2009). Proteinuria, one of the most frequent symptoms of IgAN, has been perceived as a risk factor for kidney damage in IgAN (Barbour et al., 2015)that, could accelerate the progression of ESRD (Remuzzi & Bertani, 1998). Many investigators have proven that a reduction in proteinuria can improve the prognosis of patients with IgAN (Reich et al., 2007).

Currently, the common treatments for IgAN include renin angiotensin system blockades, immunosuppressive agents, other antihypertensive agents, fish oils, anticoagulants and surgical tonsillectomy. Although there is a lack of consensus about treatment protocols due to the different clinical and pathological manifestations of IgAN, the 2012 Kidney Disease: Improving Global Outcomes (KDIGO) guidelines in 2012 (Inker et al., 2014) pointed out the importance of renin angiotensin system blockades, including angiotensin-converting-enzyme-inhibitors (ACEIs) and angiotensin-II receptor blockers (ARBs), in the treatment of proteinuria in IgAN, which would help protect kidney function by reducing proteinuria (Coppo et al., 2007b). Most importantly, the KDIGO guidelines recommended maximum supportive care, including proteinuria reduction, blood pressure control, and kidney function preservation, which remains the basis of treatment for IgA nephropathy before applying immunosuppressive agents.

Previous clinical studies and meta-analyses have shown that patients with IgAN can experience a reduction in proteinuria in response to treatment with ACEIs/ ARBs alone or a combination of ACEIs and ARBs (Remuzzi et al., 1999; Tanaka et al., 2004). However, it remains unclear which therapeutic strategy (ACEI or ARB or dual therapy) may have a better therapeutic effect on patients with IgAN in terms of a greater reduction in proteinuria and better preservation of kidney function. Thus, we conducted a Bayesian network analysis to evaluate the relative effect of these three therapeutic strategies in patients with IgAN.

## Methods

### Study selection

The protocol of this study was registered in PROSPERO, an International prospective register of systematic reviews, which is available under ID CRD42017073726.

PRISMA (PRISMA for Network Meta-Analyses) guidelines (Moher et al., 2009) were used in this study. Search strategy was similar to that described in our previous published analysis (Ye et al., 2020). PubMed, the Cochrane Library, Embase, China Biology Medicine disc, WanFang and CNKI (China National Knowledge Infrastructure) databases were searched from inception to June 2019 by a PICOS strategy without language restrictions. To identify other eligible trials, we checked the reference lists of review articles, meta-analyses, and original studies. We also searched the System for Information on Grey Literature (SIGLE), master’s and doctoral dissertations, and meeting records in the Chinese database CNKI for grey literature. We used the following search terms: “IgA nephropathy”, “proteinuria”, “albuminuria”, “microalbuminuria”, “angiotensin-receptor-blockers”, “ARBs”, “angiotensin-converting enzyme inhibitor”, “ACEI”, and the names of currently available ARBs or ACEIs (“losartan”, “valsartan”, “irbesartan”, “candesartan”, “telmisartan”, “eprosartan”, “olmesartan”, “imidapril”, “enalapril”, “lisinopril”, “captopril”, “cilazapril”, “ramipril”, “perindopril”, and “fosinopril”).

**The PICOS was as follows**:

**Population:** patients with IgA nephropathy.

**Intervention:** angiotensin-receptor-blockers, ARBs, angiotensin-converting enzyme inhibitors, ACEIs, the names of currently available ARBs or ACEIs (losartan, valsartan, irbesartan, candesartan, telmisartan, eprosartan, olmesartan, imidapril, enalapril, lisinopril, captopril, cilazapril, ramipril, perindopril, and fosinopril).

**Comparator:** angiotensin-receptor-blockers, ARBs, angiotensin-converting enzyme inhibitor, ACEIs, placebo, other antihypertensive agents.

**Outcomes**

***Primary outcomes***

Urinary protein excretion: urinary total proteinuria.

***Secondary outcomes***

Estimated glomerular filtration rate (eGFR)/blood pressure (BP).

**Study design:** RCTs.

### Inclusion and Exclusion Criteria

Studies meeting the following criteria were included: (1) randomized controlled trials (RCTs); (2) participants aged 12 years or older; and (3) participants with IgA nephropathy who reported urinary total proteinuria. Patients undergoing dialysis or kidney transplantation were excluded.

### Data extraction and outcomes of interest

Two reviewers (Zhihao Huo and Huizhen Ye) independently extracted information according to the registered protocol. The following data were extracted from each study: first author, year of publication, geographic region, and study participant characteristics (sample size, mean age, sex, duration of the intervention). Any disagreement was resolved by a third researcher (Yaozhong Kong) after a discussion.

In our study, the primary outcome was proteinuria reduction. Decreases in blood pressure and eGFR were the secondary outcomes.

### Quality assessment

We used the CASP Checklist Critical Appraisal Skills Programme (2018), an 11-question list, to make sense of the RCTs and to assess their methodological. It was made up of three sections concentrating on three problems: (1) What are the results? (2) Are the results of the study valid? (3) Will the results help locally? Only a study with more than two “Yes” answers in section A is worth proceeding with the remaining questions. In addition, we used the five-point Jadad score to assess the methodological quality of the studies, which mainly evaluated three aspects (randomization, blinding, withdrawals and dropouts) of all the studies. A score ≤2 points was defined as low quality, while a score ≥3 points was ranked as high quality.

### Data analysis

Data were analyzed as previously described in our network analysis (Ye et al., 2020). Specifically, pairwise meta-analysis and Bayesian network analysis were conducted by using ADDIS 1.16.5 software (Aggregate Data Drug Information System, The Netherlands) with a random-effects model. Heterogeneity was quantified using the I^2^ statistic, and Bayesian network analysis was conducted by using ADDIS 1.16.5 software in a Bayesian Markov chain Monte Carlo framework with a consistency model or an inconsistency model. For the ranking of the interventions, stochastic multicriteria acceptability analysis (SMAA)-based models were used (van Valkenhoef et al., 2013).

To evaluate inconsistency, we conducted node-splitting analysis and inconsistency factor with ADDIS 1.16.5 software to explore whether the direct and indirect evidence were in agreement. We could draw a conclusion with a consistency model if no relevant inconsistency existed when the 95% CIs of the random-effects standard deviation covered zero. For antiproteinuric analysis, 4 chains, including 20,000 burn-ins, 50,000 simulation iterations, 10,000 inference samples and a thinning interval of 10 for each chain, were applied. Convergence was assessed by comparing within-chain and between-chain variance to calculate the potential scale reduction factor (PSRF) (Zhao et al., 2012). It showed good convergence of iterations when the parameter “RSRF” was extremely close to 1.00. Stata MP 14.0 (64-bit) software (Computer Resource Center, USA) was used to construct a basic network diagram, showing the connections among all of the included treatments. Contribution and publication bias were also calculated with Stata MP 14.0.

**Figure 1:**
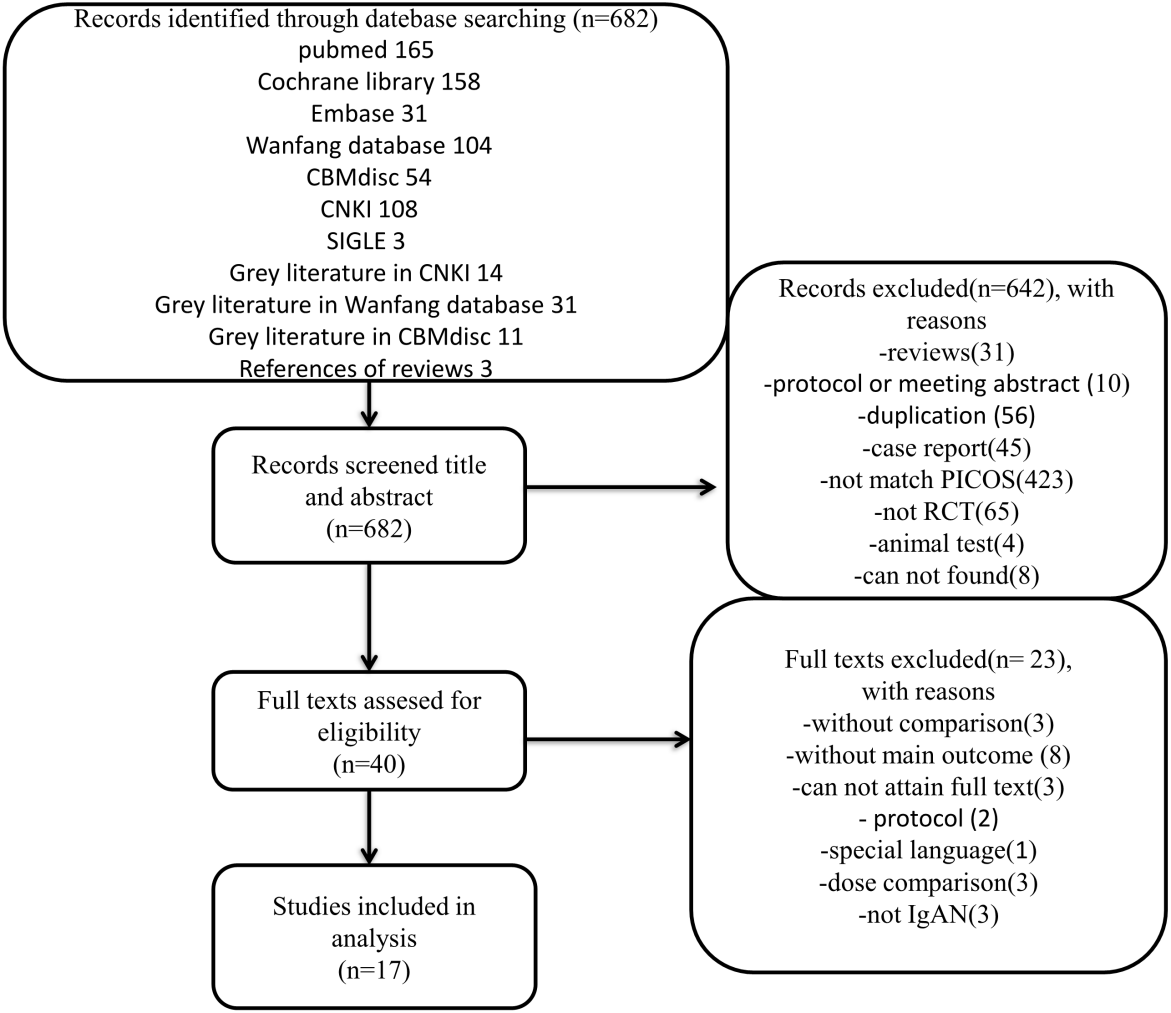
Flow diagram. Flow diagram of trial selection. CNKI, China National Knowledge Infrastructure; SIGLE, System for Information on Grey Literature; PICOS, population, intervention, comparator, outcome and study design; RCT, randomized controlled trials; IgAN, immunoglobulin A nephropathy.

We performed sample size assessment via the method called “effective sample size from an indirect comparison” recommended by Thorlund & Mills (2012).

## Results

A total of 682 records met the initial search criteria. A total of 642 articles were excluded after the title and abstract were reviewed, and 40 articles were found to be eligible for PICOS analysis. The remaining 40 articles were reviewed at the full-text level. Of these, 23 studies were excluded for various reasons, as shown in Fig. 1. Therefore, a total of 1,006 patients with IgAN in 17 RCTs published from 1994 to 2012 were suitable for inclusion and were incorporated into the analysis. A summary of the characteristics of the included studies is shown in Table 1, and the CASP checklist of the included studies is presented in Table 2.

**Figure 2:**
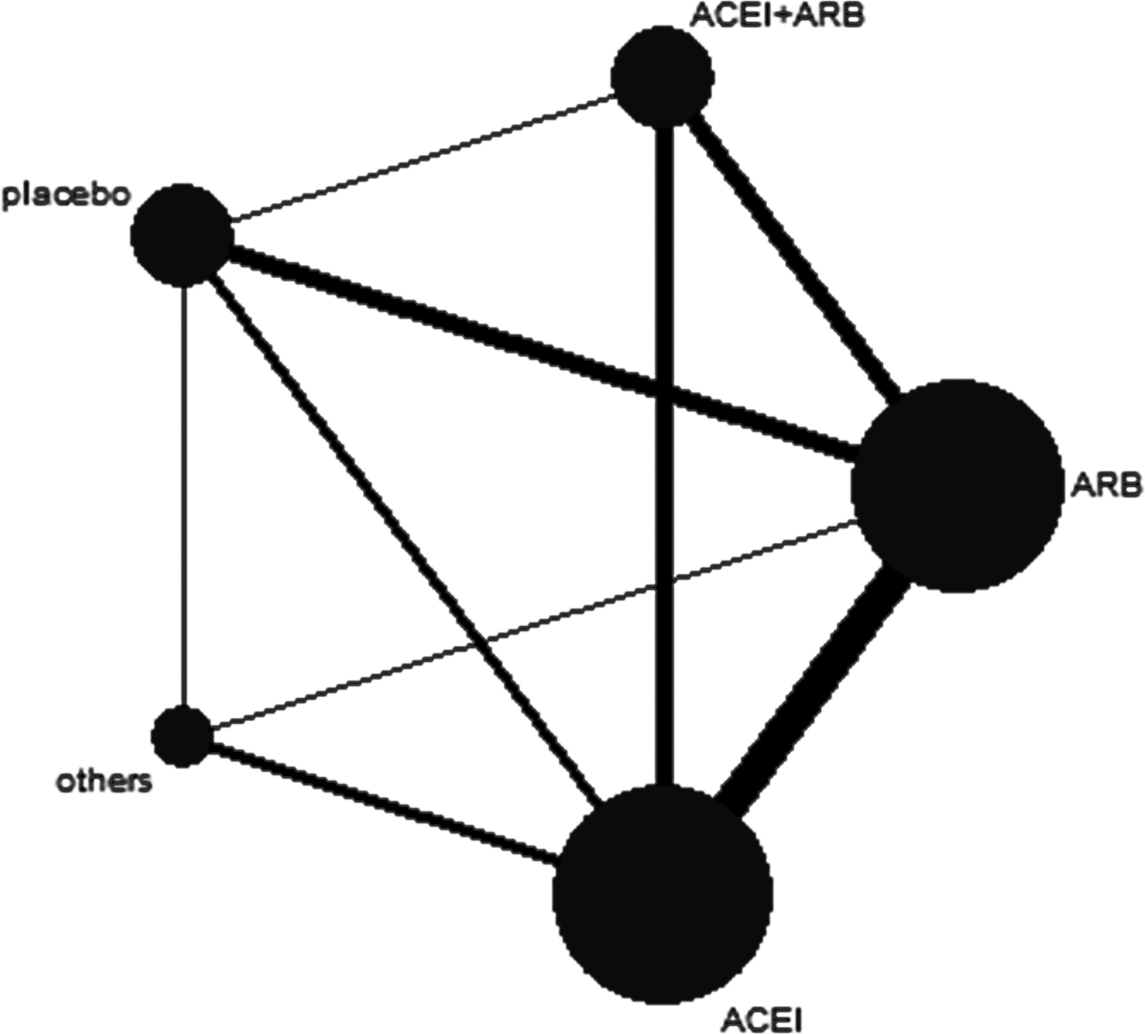
Network map. The thickness of the connecting line is proportional to the number of trials that directly compared the two medications. The size of every circle corresponds to the number of assigned patients and indicates the sample size. ACEI, angiotensin converting-enzyme inhibitor; ARB, angiotensin-II receptor blocker.

In this network study, 5 therapy regimens were considered for analysis: (1) combination therapy of ACEIs plus ARBs; (2) ACEI monotherapy; (3) ARB monotherapy; (4) other antihypertensive agents; and (5) placebo. In addition, the network map is shown in Fig. 2.

In our analysis, 528 patients were males (52.5%). Among the 17 trials, the longest follow-up period was 38 months, and 3 trials were reported to be multicenter studies.

### The consistency of the network analysis

All 95% CIs contained neutral values(zero), suggesting no evidence of inconsistency. Additionally, we conducted node-splitting analysis via direct and indirect effects as presented in Table 3, and most of the *P* values > 0.05, suggested data consistency. Hence, we conducted Bayesian network analysis with consistency random-effect models (Table 4) using ADDIS 1.16.5 software.

**Table 1:** Characteristics of included 17 RCTs.

**Reference**	**Country of origin**	**Jadad scores**	**Number for interventions**	**Interventions**	**Age (years old)**	**Sex (Male/Female)**	**N**	**Follow-up (months)**
Coppo et al. (2007a), Coppo et al. (2007b)	Italy	4	2	G1: benazepril 0.2 mg/kg qd G2: placebo	G1:21.8 ± 6.3 G2:19.3 ± 6.1	G1: 24/8 G2: 24/10	66	38
Li et al. (2006)	HK	5	2	G1: 36 patients were administered valsartan 80 mg qd, other 18 patients were administered valsartan 160 mg qd. G2: placebo	G1:40.0 ± 10.0 G2: 41.0 ± 9.0	G1: 13/41 G2: 17/38	109	26
Shi et al. (2002)	China	2	2	G1: benazepril 10 mg qd G2: CCB, *α* receptor blocker and/or *β* receptor blocker	G1: 12 to 53 G2: 12 to 72	G1: 47/18 G2: 40/26	131	18
Horita et al. (2004)	Japan	2	3	G1: temocapril 1 mg qd G2: losartan 12.5 mg qd G3: temocapril 1 mg+losartan 12.5 mg qd	G1: 39.6 ± 10.8 G2: 42.7 ± 12.0 G3: 39.6 ± 10.4	G1: 4/6 G2: 5/5 G3: 5/6	31	6
Horita et al. (2006)	Japan	2	3	G1: temocapril 1 mg qd G2: losartan 12.5 mg qd G3: temocapril 1 mg+losartan 12.5 mg qd	G1: 43.3 ± 10.9 G2: 42.9 ± 12.2 G3: 38.0 ± 9.2	G1: 8/6 G2: 9/7 G3: 7/6	43	12
Tanaka et al. (2004)	Japan	2	2	G1: enalapril 0.1 mg/kg qd (up to 5 mg qd) and losartan 1 mg/kg qd (up to 50 mg) G2: without those agents	G1: 12.3 ± 2.0 G2: 12.3 ± 2.0	G1: 2/2 G2: 3/2	9	24
Praga et al. (2003)	Spain	3	2	G1: enalapril 5 mg qd G2: other antihypertensive drugs	G1: 27.8 ± 12.0 G2: 29.9 ± 12.3	G1: 15/8 G2: 12/9	44	78
Remuzzi et al. (1999)	Italy	3	2	G1: enalapril 20 mg qd G2: irbesantan 100 mg qd	G1: 20 to 65 G2: 20 to 65	NG	20	1
Kanno et al. (2005)	Japan	2	2	G1: temocapril or trandolapril 1–2 mg qd G2: amlodipine 2.5–5 mg qd	G1: 35 ± 2 G2: 35 ± 3	G1: 8/18 G2: 12/11	49	36
Park et al. (2003)	Korea	2	2	G1: losartan 50 mg qd G2: amlodipine 5 mg qd	G1: 39.3 ± 8.7 G2: 44.3 ± 13.4	G1: 9/11 G2:9/7	36	12
Perico et al. (1998)	Italy	3	2	G1: enalapril 20 mg qd G2: irbesartan 100 mg qd	G1: 31(20-54) G2: 46(34–65)	G1:9/2 G2:7/2	20	1
Shimizu et al. (2008)	Japan	2	2	G1: losartan 12.5 mg qd G2: placebo	G1: 36.0 ± 8.5 G2: 35.7 ± 8.1	G1: 11/7 G2: 6/12	36	12
Maschio et al. (1994)	Italy	3	2	G1: fosinopril 20 mg qd G2: placebo	NG	NG	78	8
Nakamura et al. (2007)	Japan	4	3	G1: olmesartan 10 mg qd G2: temocapril 2 mg qd G3:olmesartan 10 mg+ temocapril 2 mg qd	G1: 34 ± 7 G2: 31 ± 8 G3: 31 ± 7	G1: 5/3 G2: 4/4 G3: 4/4	24	3
Renke et al. (2004)	Poland	2	3	G1: losartan 25 mg qd G2: enalapril 10 mg qd G3: losartan 25 mg+enalapril 10 mg qd	G1: 40.4 ± 11.9 G2: 43.4 ± 10.1 G3: 37.7 ± 12.7	G1: 7/11 G2: 12/6 G3: 11/5	52	9
Shen et al. (2012)	China	3	2	G1: losartan 50 mg qd G2: placebo	G1: 50.2 ± 10.4 G2: 49.1 ± 11.5	G1: 58/54 G2: 56/58	226	12
Nakamura et al. (2000)	Japan	2	4	G1: verapamil 120 mg qd G2: trandolapril 2 mg qd G3: candesartan cilexetil 8 mg qd G4: placebo	NG	NG	32	3

**Notes.**

NG not given G1 Group 1 G2 Group 2 G3 Group 3 G4 Group 4

Values are mean ±[SD].

**Table 2:** CASP checklist of included 17 RCTs.

**Reference**	**Section A**						**Section B**		**Section C**			
	**Q1**	**Q2**	**Q3**	**Q4**	**Q5**	**Q6**	**Q7 (1.what outcomes were measured ?)**	**2. (Is the primary outcome clearly specified ?)**	**Q8**	**Q9**	**Q10**	**Q11**
Coppo et al. (2007b)	Y	Y	Y	Y	Y	Y	proteinuria, eGFR, SBP, DBP, MAP	Y	NG	Y	Y	Y
Li et al. (2006)	Y	Y	Y	Y	Y	Y	proteinuria, eGFR, SBP, DBP, MAP, serum creatinine	Y	Y	Y	Y	Y
Shi et al. (2002)	Y	Y	Y	N	Y	Y	proteinuria, eGFR, MAP, serum creatinine	Y	NG	Y	Y	Y
Horita et al. (2004)	Y	Y	Y	Y	Y	Y	proteinuria, SBP, DBP, MAP, serum creatinine, serum total protein	Y	NG	Y	Y	Y
Horita et al. (2006)	Y	Y	Y	Y	Y	Y	UPE, eGFR, SBP, DBP, serum creatinine, PAC, PRA, BUN	Y	NG	Y	Y	Y
Tanaka et al. (2004)	Y	Y	Y	N	Y	Y	proteinuria, serum creatinine	Y	N	Y	Y	Y
Praga et al. (2003)	Y	Y	Y	NG	Y	Y	proteinuria, eGFR, MBP, serum creatinine	Y	Y	Y	Y	Y
Remuzzi et al. (1999)	Y	Y	Y	Y	Y	Y	proteinuria, eGFR, SBP, DBP, MAP	Y	NG	Y	Y	Y
Kanno et al. (2005)	Y	Y	Y	NG	Y	Y	proteinuria, eGFR, SBP, DBP, serum creatinine	Y	NG	Y	Y	Y
Park et al. (2003)	Y	Y	Y	NG	Y	Y	proteinuria, eGFR, SBP, DBP, MAP, TGF- *β*1 excretions, uric acid	Y	NG	Y	Y	Y
Perico et al. (1998)	Y	Y	Y	Y	Y	Y	proteinuria, eGFR, SBP, DBP, MAP, serum creatinine, serum total protein	Y	NG	Y	Y	Y
Shi et al. (2002)	Y	Y	Y	N	Y	Y	proteinuria, eGFR, SBP, DBP, serum creatinine, serum uric acid	Y	NG	Y	Y	Y
Maschio et al. (1994)	Y	N	Y	Y	NG	Y	proteinuria, eGFR, MAP	Y	NG	Y	Y	Y
Nakamura et al. (2007)	Y	Y	Y	N	Y	Y	proteinuria, eGFR, serum creatinine, L-FABP,8-OHdG	Y	NG	Y	Y	Y
Renke et al. (2004)	Y	Y	Y	N	Y	Y	proteinuria, SBP, DBP, serum creatinine	Y	NG	Y	Y	Y
Shen et al. (2012)	Y	Y	Y	N	Y	Y	proteinuria, eGFR, SBP, DBP, serum creatinine, serum uric acid	Y	NG	Y	Y	Y
Nakamura et al. (2000)	Y	Y	Y	NG	Y	Y	proteinuria, eGFR, serum creatinine, BUN, number of urinary podocytes	Y	NG	Y	Y	Y

**Notes.**

Q1: Did the trial address a clearly focused issue? Q2: Was the assignment of patients to treatments randomised? Q3: Were all of the patients who entered the trial properly accounted for at its conclusion? Q4: Were patients, health workers and study personnel ‘blind’ to treatment? Q5: Were the groups similar at the start of the trial? Q6: Aside from the experimental intervention, were the groups treated equally? Q7: How large was the treatment effect? Q8: How precise was the estimate of the treatment effect? Q9: Can the results be applied to the local population, or in your context? Q10: Were all clinically important outcomes considered? Q11:Are the benefits worth the harms and costs?

Y yes N no NG not given eGFR estimated Glomerular filtration rate SBP systolic blood pressure DBP diastolic blood pressure MAP Mean arterial pressure UPE urinary protein excretion PAC plasma aldosterone concentration PRA plasma renin activity BUN blood urea nitrogen L-FABP Liver-type fatty acid-binding protein 8-OHdG 8-hydroxydeoxyguanosine

**Table 3:** Node-splitting analysis of proteinuria reduction.

**Interventions**	**Direct Effect**	**Indirect Effect**	**Overall**	*P*-Value
ACEI vs ACEI+ARB	0.37 (−0.02, 0.80)	0.11 (−0.84, 1.07)	0.32 (−0.03, 0.69)	0.60
ACEI vs ARB	0.10 (−0.19, 0.41)	−0.12 (−0.73, 0.46)	0.04 (−0.22, 0.33)	0.49
ACEI vs Placebo	−0.49 (−0.88, −0.10)	−0.33 (−0.80, 0.16)	−0.46 (−0.76, −0.15)	0.60
ACEI+ARB vs ARB	−0.25 (−0.68, 0.15)	−0.34 (−0.84, 0.16)	−0.28 (−0.63, 0.08)	0.77
ACEI+ARB vs Placebo	−0.61 (−1.50, 0.30)	−0.82 (−1.31, −0.37)	−0.77 (−1.20, −0.38)	0.65
ARB vs Other Antihypertensive Agents	−0.24 (−0.92, 0.41)	−1.48 (−2.05, −0.93)	−0.87 (−1.39, −0.38)	0.01
ARB vs Placebo	−0.52 (−0.90, −0.11)	−0.40 (−0.91, 0.07)	−0.50 (−0.82, −0.20)	0.68
Others vs Placebo	−0.38 (−1.18, 0.38)	0.55 (0.01, 1.09)	0.36 (−0.14, 0.90)	0.05

**Notes.**

Values are mean ±[SD].

Direct effects refer to the summary of direct effects for each split comparison.

Indirect effects refer to the summary of the indirect effects for each split comparison.

*P*-values refer to inconsistency *p*-values for each split comparison.

**Table 4:** Outcomes of ranking from all RCTs.

	**Proteinuria reduction**	**BP reduction**	**eGFR reduction**
ACEI+ARB	1(92%)	1(92%)	1(92%)
ARB	2(51%)	2(53%)	2(52%)
ACEI	3(53%)	3(55%)	3(54%)
Placebo	4(89%)	4(89%)	4(88%)
Other Antihypertensive Agents	5(89%)	5(89%)	5(88%)

**Notes.**

For Proteinuria reduction, rank 1 is best, rank N is worst.

For BP reduction, rank 1 is best, rank N is worst.

For eGFR reduction, rank N is best, rank 1 is worst.

Values are ranking number (rank probability).

### Results of the meta-analysis

Table 5 showed the meta-analysis results for proteinuria. Four studies reported the effects of combination therapy of ACEI plus ARB vs ACEI monotherapy on proteinuria. There was a significant difference between the two groups, with SMD = −1.04 (95% CI [−1.47 to −0.62]). Data on the antiproteinuric effect of ACEI plus ARB vs ARB monotherapy were available in 4 studies, which achieved a significant difference between the two groups, with SMD = −0.67 (95% CI [−1.06 to −0.27]). Nine studies reported on the antiproteinuric effects of ACEI therapy regimens vs ARB therapy regimens. There was no significant difference in reducing proteinuria, with SMD = 0.14 (95% CI [−0.37 to −0.65]).

**Table 5:** Response rates for efficacy in meta-analyses of direct comparisons between each pair of drugs.

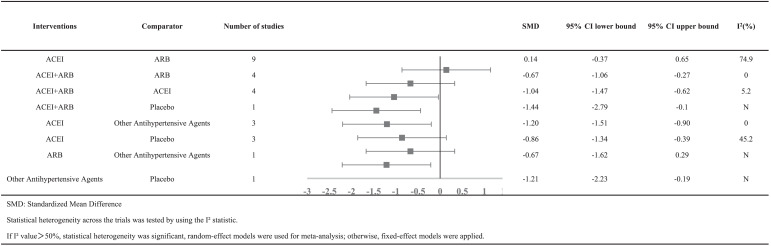

### Results of the Bayesian network analysis

Proteinuria reduction was reported in all 17 trials (Coppo et al., 2007b; Horita et al., 2004; Horita et al., 2006; Kanno et al., 2005; Li et al., 2006; Maschio et al., 1994; Nakamura et al., 2007; Nakamura et al., 2000; Park et al., 2003; Perico et al., 1998; Praga et al., 2003; Remuzzi et al., 1999; Renke et al., 2004; Shen et al., 2012; Shi et al., 2002; Shimizu et al., 2008; Tanaka et al., 2004). The primary outcome was ranked as ACEI plus ARB > ARB > ACEI  > placebo > other antihypertensive agents, according to the Bayesian probability framework. The combination therapy of ACEIs and ARBs appeared to have a significantly more antiproteinuric effect (92%) in IgAN patients, followed by ARB monotherapy in second place (51%) and ACEI therapy regimens ranking third (53%).

Among the 17 included trials, systolic blood pressure reduction was reported in 9 trials (Coppo et al., 2007b; Horita et al., 2004; Horita et al., 2006; Kanno et al., 2005; Nakamura et al., 2000; Perico et al., 1998; Remuzzi et al., 1999; Renke et al., 2004; Shen et al., 2012). The ranks of the decrease in blood pressure were ACEI plus ARB >ARB > ACEI > placebo > other antihypertensive agents. It seemed that co-administration of ACEIs and ARBs was most likely to rank first (92%) in terms of the greatest reduction in blood pressure, followed by ARB monotherapy in second place (53%) and ACEI therapy regimens ranking third (55%).

In addition, a total of 14 studies (Coppo et al., 2007b; Horita et al., 2004; Horita et al., 2006; Kanno et al., 2005; Li et al., 2006; Maschio et al., 1994; Nakamura et al., 2000; Park et al., 2003; Perico et al., 1998; Praga et al., 2003; Remuzzi et al., 1999; Shen et al., 2012; Shi et al., 2002; Shimizu et al., 2008) were included for the network analysis of eGFR reduction. The ranks of the magnitude of kidney function decline were ACEI plus ARB >ARB > ACEI > placebo > other antihypertensive agents. In other words, rank first means the largest decline in eGFR, resulting in the worst kidney function. The combination therapy of ACEIs and ARBs seemed to rank first (92%), with ARB monotherapy ranking second (52%) and ACEI therapy regimens ranking third (54%).

### Sensitivity analysis

To assess any impact of study quality on the effect estimates, a sensitivity analysis was also conducted. Of these 17 studies, considering that different stages of chronic kidney disease may reduce the accuracy of the results, 2 studies were excluded. One was because of eGFR < 60 ml/min/1.73 m^2^, and the other was due to not reporting the eGFR before treatment. Finally, the remaining 15 studies were included in the sensitivity analysis. No change was observed in the ranking of the antiproteinuric effects. For kidney function protection, the study of Shen PC et al. was excluded in the sensitivity analysis for low eGFR baseline (Shen et al., 2012), while four studies were excluded because of dropouts or missing data. Thus, 12 studies were included, and no change was observed in the ranking of kidney function protection. In other words, there was still the highest possibility for ACEIs to be the most renoprotective therapy regimens.

### Publication bias

**Figure 3:**
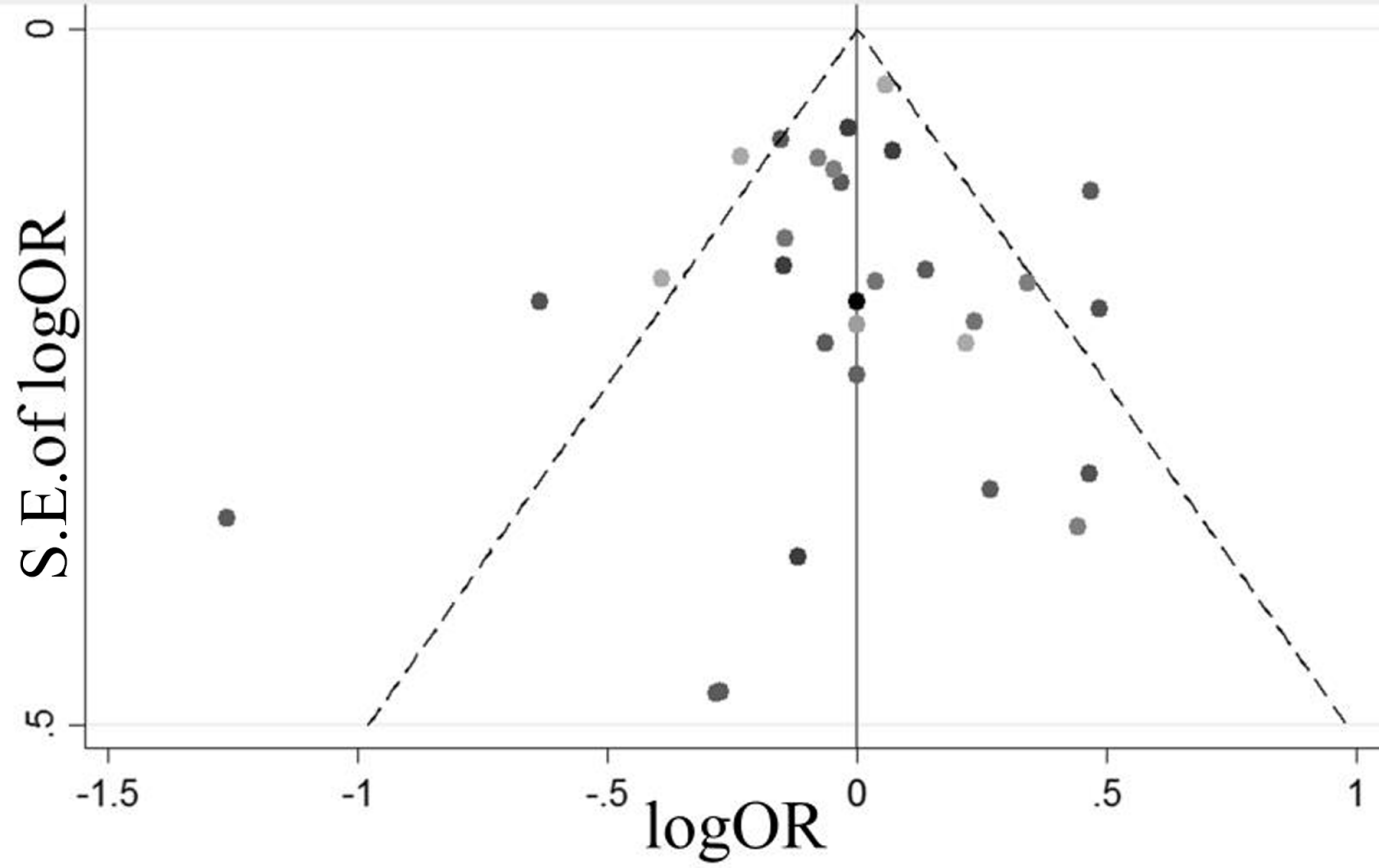
A funnel plot of all the studies.

Figure 3 presents a funnel plot of all of the studies. We found that all studies fell inside the 95% CIs and were distributed around the vertical direction, indicating no obvious publication bias.

### Sample size assessment

To explore the effective sample size, we performed sample size assessment according to “Sample size and power considerations in network” (Thorlund & Mills, 2012). The results suggested that the number of patients between treatment ACEIs plus ARBs therapy vs. ACEI should be 101 in total. In fact, the numbers were far below the actual numbers included in our study, revealing that the sample size in our study was adequate.

## Discussion

This Bayesian network analysis showed that combination therapy of ACEIs plus ARBs significantly seemed to have the best antiproteinuric effect and a greater reduction of blood pressure in patients with IgA nephropathy compared with single drug regimens. In addition, ACEIs appeared to be the best therapeutic approach for kidney repair, although ARBs were more likely to reduce proteinuria than ACEI therapy.

Proteinuria, an indispensable risk factor for the progression of IgAN, could be reduced by renin angiotensin system blockades, and its remission could improve the prognosis of patients with IgAN (Reich et al., 2007). Our study found that a combination of ACEIs and ARBs could exert an additive antiproteinuric effect, which is in accordance with previous studies (Bhattacharjee & Filler, 2002; Dillon, 2004; Horita et al., 2004; Horita et al., 2006; Nakamura et al., 2007; Tanaka et al., 2004). The reasons for this may be as follows. First, dual drug treatment could hamper angiotensin II’s (Ang II) effects on intrarenal hemodynamics more extensively by decreasing glomerular capillary pressure and thereby ameliorate glomerular hyperfiltration. It is acknowledged that the binding of Ang II to the angiotensin type-1 receptors (AT1R) can cause vasoconstriction, enhanced sympathetic nervous system activity and increased sodium retention, which can lead to high blood pressure (Ames, Atkins & Pitt, 2019). ACEIs inhibit the transformation of Ang I to Ang II, while ARBs selectively prevent the binding of Ang II to AT1R (Zhang et al., 2020). In addition, Ang II can act independently of angiotensin-converting-enzyme (ACE), such as via human chymase tonin and cathepsin G (Mento & Wilkes, 1987; Phillips, Speakman & Kimura, 1993; Urata et al., 1993). Therefore, dual therapy reflects two distinct mechanisms for reducing proteinuria and blood pressure more efficiently: acting on Ang II synthesis and Ang II receptors. Second, dual therapy regimens are thought to be a good way to improve glomerular permselectivity (Remuzzi et al., 1999; Woo et al., 2000). Third, some investigators found that the ACEI/ARB combination could alleviate glomerular and tubulointerstitial injury because of the resulting reduction in the L-FABP and ET-1 levels, which are correlated with tubulointerstitial lesions and renal fibrosis (Kamijo et al., 2005; Nakamura et al., 2007). Fourth, it was reported that combining ACEIs and ARBs can inhibit the synthesis and secretion of renal TGF- *β*1 (Scaglione et al., 2005; Song et al., 2003), which is thought to contribute to the natural course of human IgA glomerulonephritis (Haramaki et al., 2001; Niemir et al., 1995; Yamamoto et al., 1996). Thus, reducing TGF- *β*1 means slowing down the natural course of ESRD for IgAN patients.

Collectively, co-administration of ACEIs and ARBs have greater inhibition of the adverse effects made of Ang II. Admittedly, reducing blood pressure and proteinuria to a certain extent can preserve kidney function. However, it does not mean that this can translate into a better outcome with eGFR in the long run because the decline in eGFR is also related to many factors, such as hyperglycemia, hypoproteinemia, anemia, smoking, hyperlipidemia, hyperhomocysteinemia, advanced age, malnutrition, and uremic toxin. Additionally, it is noteworthy that combining the ACEIs and ARBs would magnify side effects such as hyperkalemia and acute kidney injury (Fried et al., 2013). Joint National Committee (JNC 8) Guidelines (James et al., 2014) stress that the use of an ACEI and an ARB together in the same patient is not advocated due to their side effects of increased serum creatinine and a greater possibility of causing hyperkalemia than monotherapy. The 2012 KDIGO Clinical Practice Guideline for the Evaluation and Management of CKD (Inker et al., 2014) also disagreed with the combination of ACEI plus ARB therapy because of obvious side effects, such as hyperkalemia, hypotension and AKI. Therefore, we nephrologists ought to weigh the pros and cons and safety and effectiveness when choosing dual blockade of the renin-angiotensin-aldosterone system (RAAS), and we recommend that the levels of serum creatinine, potassium and blood pressure should be closely monitored during combination drug use.

In the present network study, the second major finding was that ACEIs seemed to be a more appropriate choice to restore kidney function than dual therapy or ARB monotherapy in IgAN. The results of this study are consistent with those of others (Cattran, Greenwood & Ritchie, 1994; Feriozzi et al., 1989; Kanno et al., 2005; Praga et al., 2003). First, the main mechanism of ACEI therapy is to ameliorate nephrotic hemodynamics by reducing the adverse effects of Ang II directly, which is the arch criminal in kidney fibrosis (Coppo et al., 2007a). Second, increasing evidence indicates that ACEIs could attenuate oxidative stress by scavenging oxygen free radicals and therefore improve endothelial dysfunction and exert a renoprotective effect (Nakamura et al., 2007; Yasunari et al., 2004). In addition, Hayata et al. found that attenuating oxidative stress, which is involved in the process of developing kidney interstitial fibrosis, leads to slowing of the progression of IgAN patients to ESRD (Hayata et al., 2012). Recently, Fang and his colleagues (Fang et al., 2018) proved that ACEIs could attenuate scar formation by suppressing TGF- *β*1. Therefore, long-term treatment with ACEI therapy shows beneficial effects in protecting kidney function. Zhang GH and Hou FFs study (Zhang et al., 2005) also revealed that ACEI monotherapy still slows the progression of CKD in patients with a Scr higher than 266 micromol/L, and it also shows good renoprotective effects. In our analysis, it is noteworthy that the low eGFR in the group with dual RAS blockade may perhaps be due to faster disease progression and less renoprotection. On the other hand, it is difficult to exclude that most of the effects on eGFR are reversible.

Recently, Lennartz et al. based on the results of the 3-year trial STOP-IgAN, found no obvious difference in blood pressure between treatment groups, but patients on dual RAS blockade had a slightly higher level of proteinuria. In addition, there was no significant difference between groups regarding the loss of kidney function during the trial (Lennartz et al., 2020). The results of this article differ from our analysis mainly because of the differences in the methods. First, the intervention conditions in this article were different from those in our analysis. Participants in the STOP-IgAN trial were randomized into the 3-year trial phase and were assigned to either continue supportive therapy alone or to receive additional immunosuppression after a 6-month run-in phase with comprehensive optimization of supportive treatment strategies. In addition, a distinction between different ACE inhibitors or ARB substances was not made in the group with single RAS blockade. However, only IgA nephropathy patients with specific ACEIs or ARBs or their combination were included in our network meta-analysis. Moreover, full clinical remission and eGFR loss ≥15 ml/min/1.73 m^2^ were the primary endpoints of the STOP-IgAN trial, while urinary total proteinuria was our primary outcome.

However, some limitations in the present network analysis should be considered. First, the inclusion criteria regarding blood pressure in the trials were different. Current therapeutic guidelines (Inker et al., 2014) recommend BP values < 130/80 mmHg in patients with proteinuria>0.3 g/d. We have no idea if strict control of BP to ≤130/80 mmHg might have changed the results. However, in agreement with many investigators (Coppo et al., 2007b; Hemmelder, De Zeeuw & De Jong, 1999; Nakamura et al., 2007; Nakamura et al., 2000; Praga et al., 2003), renin angiotensin system blockades could exert an antiproteinuric effect independent of a reduction in blood pressure. Second, there might be an effect on the conclusions because the studies we included did not all use the same ratio or dosage in their treatments. Third, the conclusion cannot be applied to IgA nephropathy with a long course because the duration of the 17 included RCTs was relatively short, with an average follow-up of 17.6 months. Fourth, our network analysis did not evaluate concerns about polymorphisms of the ACE gene, or the AT1R gene, which are associated with glomerular disease susceptibility, natural history, and the response to therapy. Yoshida et al. (1995) revealed a high frequency of the DD genotype in patients with IgAN, and found its presence was associated with progressive renal deterioration and it was also associated with a higher antiproteinuric response to ACE inhibition (Dillon, 2004).

## Conclusions

This network meta-analysis indicates that a combination of ACEIs and ARBs seems to have a significantly better antiproteinuric effect and a greater reduction of blood pressure than ACEI or ARB monotherapy for IgA nephropathy. However, we recommend that the levels of serum creatinine, potassium and blood pressure should be closely monitored during combination drug use because of the potential harms. Our findings also imply that ACEIs would have the highest probability of protecting kidney function among all three therapies. Additional large, well-designed RCTs with longer follow-up periods are warranted to confirm these findings.

##  Supplemental Information

10.7717/peerj.11661/supp-1 Supplemental Information 1 Raw data

10.7717/peerj.11661/supp-2 Supplemental Information 2 PRISMA checklist

10.7717/peerj.11661/supp-3 Supplemental Information 3 The Rationale for Conducting the Meta-analysis

10.7717/peerj.11661/supp-4 Supplemental Information 4 Protocols

10.7717/peerj.11661/supp-5 Supplemental Information 5 Rationale for Netwok Meta-analysis This document explains the position of network meta-analysis. Bayesian network analysis is an extension of traditional meta-analysis. It has allowed the estimation of metrics for all possible comparisons in the same model, simultaneously gathering direct and indirect evidence, while traditional systematic reviews and pairwise meta-analyses usually compares only two interventions at the time. How to choose suitable therapeutic strategies is a common clinical problem, but no clinical studies have focused on all of these therapies. Thus, here, we tried to offer a reference to clinical practitioners.

## Abbreviations

IgAN: immunoglobulin A nephropathy
ACEI: angiotensin-converting-enzyme-inhibitor
ARB: angiotensin-II receptor blocker
CNKI: China National Knowledge Infrastructure
CKD: chronic kidney disease
ESRD: end-stage renal disease
RCTs: randomized controlled trials
PRISMA: Preferred Reporting Items of Systematic Reviews and Meta-Analyses
SIGLE: System for Information on Grey Literature
eGFR: estimated glomerular filtration rate
BP: blood pressure
AKI: acute kidney injury
KDIGO: kidney disease improving global outcomes
AT1R: angiotensin type-1 receptors
RAAS: renin-angiotensin-aldosterone system

## Additional Information and Declarations

### Data Availability

The following information was supplied regarding data availability: The raw data are available in the Supplemental File.
