# Supplementary material for: Comparative efficacy of different renin angiotensin system blockade therapies in patients with IgA nephropathy: a Bayesian network meta-analysis of 17 RCTs
Source: PeerJ. 2021 Jul 6;9:e11661. doi: 10.7717/peerj.11661 (PMC8269645; doi:10.7717/peerj.11661)
Supplement: Supplemental Information 3 [file peerj-09-11661-s003.docx]

Supplemental Files:

1.The rationale for conducting the meta-analysis

Response: The objective of this article is to evaluate the relative effects of different therapeutic strategies (ACEI or ARB or their combination) in patients with IgAN results in more reduction of proteinuria and better preservation of kidney function. However, the lack of original head-to-head direct trials laid barriers on this study.

Bayesian network analysis is an extension of traditional meta-analysis, which can make indirect comparisons of two treatments through a common comparator in the absence of head-to-head direct trials. Therefore, we performed a Bayesian network analysis to evaluate the relative proteinuria reduction and renal function changes by various ACEIs or ARBs or their combination for patients with IgAN.

Network meta-analysis, is an indirect treatment comparison technique (NMA), to compare two treatments in the situation where an indirect comparison between two treatments of interest can be obtained through more than one common comparator or linking treatment. For instance, consider a setting where there is interest in performing an indirect comparison between treatment A and treatment B. If trials have separately compared treatment A to C, treatment B to C, treatment A to D, and treatment B to D, this method allows investigators to incorporate results from trials in which the common comparator was C, as well as trials in which the common comparator was D. Thus, more than one common treatment can be used to conduct an indirect comparison between two treatments. NMA also allows determining the amount of agreement between the results obtained when different linking treatments are used. More details can be obtained in supplemental file named “rationale for network meta-analysis”.

2. The contribution that the meta-analysis makes to knowledge in light of previously published related reports, including other meta-analyses and systematic reviews.

Response:

Previous studies have testified that patients with IgAN can get a reduction of proteinuria by the treatment of ACEI/ ARB alone or a combination of ACEIs and ARBs (Remuzzi et al. 1999; Tanaka et al. 2004). A clinical study by Nakamura T (Nakamura et al. 2000) reported that trandolapril (ACEI) and candesartan colexetil (ARB) effected a similar reduction in proteinuria in IgAN, while other studies (Dillon 2004; Horita et al. 2004; Horita et al. 2006; Nakamura et al. 2007; Tanaka et al. 2004) revealed that combination of ACEI and ARB could exert a more antiproteinuric effect than monotherapy. However, it remains unclear now what should be recommended for more proteinuria reduction and less renal function impairment in patients with IgAN.

A recent network meta-analysis by Huang R et al.(Huang et al. 2017) reported that the ACEI-ARB combination therapy of trandolapril+candesartan was the most efficacious in reducing albuminuria for normotensive diabetic patients. But the study only included the diabetic patients and did not take renal function fluctuation into consideration, which means that the results cannot be generalized to IgAN patients. It also did not report the effects on blood pressure reduction, which is important for clinical practitioners. Thus, we were going to conduct a Bayesian network analysis to evaluate the relative effects of these three therapeutic strategies (ACEIs, ARBs and their combination) in patients with IgAN.

**Reference**

Remuzzi A, Perico N, Sangalli F, Vendramin G, Moriggi M, Ruggenenti P, and Remuzzi G. 1999. ACE inhibition and ANG II receptor blockade improve glomerular size-selectivity in IgA nephropathy. *Am J Physiol* 276:F457-466. 10.1152/ajprenal.1999.276.3.F457

Tanaka H, Suzuki K, Nakahata T, Tsugawa K, Konno Y, Tsuruga K, Ito E, and Waga S. 2004. Combined therapy of enalapril and losartan attenuates histologic progression in immunoglobulin A nephropathy. *Pediatr Int* 46:576-579. 10.1111/j.1442-200x.2004.01955.x

Nakamura T, Ushiyama C, Suzuki S, Hara M, Shimada N, Sekizuka K, Ebihara I, and Koide H. 2000. Effects of angiotensin-converting enzyme inhibitor, angiotensin II receptor antagonist and calcium antagonist on urinary podocytes in patients with IgA nephropathy. *Am J Nephrol* 20:373-379. 10.1159/000013619

Huang R, Feng Y, Wang Y, Qin X, Melgiri ND, Sun Y, and Li X. 2017. Comparative Efficacy and Safety of Antihypertensive Agents for Adult Diabetic Patients with Microalbuminuric Kidney Disease: A Network Meta-Analysis. *PLoS One* 12:e0168582. 10.1371/journal.pone.0168582

Dillon JJ. 2004. Angiotensin-converting enzyme inhibitors and angiotensin receptor blockers for IgA nephropathy. *Semin Nephrol* 24:218-224.

Horita Y, Tadokoro M, Taura K, Suyama N, Taguchi T, Miyazaki M, and Kohno S. 2004. Low-dose combination therapy with temocapril and losartan reduces proteinuria in normotensive patients with immunoglobulin a nephropathy. *Hypertens Res* 27:963-970.

Horita Y, Taura K, Taguchi T, Furusu A, and Kohno S. 2006. Aldosterone breakthrough during therapy with angiotensin-converting enzyme inhibitors and angiotensin II receptor blockers in proteinuric patients with immunoglobulin A nephropathy. *Nephrology (Carlton)* 11:462-466. 10.1111/j.1440-1797.2006.00665.x

Nakamura T, Inoue T, Sugaya T, Kawagoe Y, Suzuki T, Ueda Y, Koide H, and Node K. 2007. Beneficial effects of olmesartan and temocapril on urinary liver-type fatty acid-binding protein levels in normotensive patients with immunoglobin A nephropathy. *Am J Hypertens* 20:1195-1201. 10.1016/j.amjhyper.2007.06.003

Tanaka H, Suzuki K, Nakahata T, Tsugawa K, Konno Y, Tsuruga K, Ito E, and Waga S. 2004. Combined therapy of enalapril and losartan attenuates histologic progression in immunoglobulin A nephropathy. *Pediatr Int* 46:576-579. 10.1111/j.1442-200x.2004.01955.x
