# Supplementary material for: Comparative efficacy of different renin angiotensin system blockade therapies in patients with IgA nephropathy: a Bayesian network meta-analysis of 17 RCTs
Source: PeerJ. 2021 Jul 6;9:e11661. doi: 10.7717/peerj.11661 (PMC8269645; doi:10.7717/peerj.11661)
Supplement: Supplemental Information 4 [file peerj-09-11661-s004.pdf]

UNIVERSITY of York
Centre for Reviews and Dissemination

NHS National Institute for Health Research

PROSPERO International prospective register of systematic reviews

Review title and timescale

1 **Review title**
Give the working title of the review. This must be in English. Ideally it should state succinctly the interventions or exposures being reviewed and the associated health or social problem being addressed in the review.
We are going to conduct a Bayesian network analysis to evaluate the relative effect of different renin angiotensin system blockades methods, including angiotensin receptor-blockers(ARB), angiotensin-converting enzyme inhibitor(ACEI) and ACEI+ARB in patients with IgA nephropathy.

2 **Original language title**
For reviews in languages other than English, this field should be used to enter the title in the language of the review. This will be displayed together with the English language title.

3 **Anticipated or actual start date**
Give the date when the systematic review commenced, or is expected to commence.
16/09/2017

4 **Anticipated completion date**
Give the date by which the review is expected to be completed.
17/09/2018

5 **Stage of review at time of this submission**
Indicate the stage of progress of the review by ticking the relevant boxes. Reviews that have progressed beyond the point of completing data extraction at the time of initial registration are not eligible for inclusion in PROSPERO. This field should be updated when any amendments are made to a published record.

The review has not yet started √

Review stage	Started	Completed
Preliminary searches	No	No
Piloting of the study selection process	No	No
Formal screening of search results against eligibility criteria	No	No
Data extraction	No	No
Risk of bias (quality) assessment	No	No
Data analysis	No	No

Provide any other relevant information about the stage of the review here.

Review team details

6 **Named contact**
The named contact acts as the guarantor for the accuracy of the information presented in the register record.
Huizhen Ye

7 **Named contact email**
Enter the electronic mail address of the named contact.
455108698@qq.com

8 **Named contact address**
Enter the full postal address for the named contact.
528000

9 **Named contact phone number**
Enter the telephone number for the named contact, including international dialing code.
15902039011

10 **Organisational affiliation of the review**
Full title of the organisational affiliations for this review, and website address if available. This field may be completed as 'None' if the review is not affiliated to any organisation.
None
Website address:

11 **Review team members and their organisational affiliations**
Give the title, first name and last name of all members of the team working directly on the review. Give the organisational affiliations of each member of the review team.

Title	First name	Last name	Affiliation
Dr	Huizhen	Ye	Nephrology Department, The First People's

Hospital of Foshan, FoShan, Guangdong, China

Professor	Yaozhong	Kong	Nephrology Department, The First People's Hospital of Foshan, FoShan, Guangdong, China
Ms	Peiyi	Ye	Nephrology Department, The First People's Hospital of Foshan, FoShan, Guangdong, China
Dr	Zhe	Zhang	Nephrology Department, The First People's Hospital of Foshan, FoShan, Guangdong, China
Dr	Zhihao	Huo	Nephrology Department, The First People's Hospital of Foshan, FoShan, Guangdong, China
Dr	Wei	Li	Nephrology Department, The First People's Hospital of Foshan, FoShan, Guangdong, China

12 Funding sources/sponsors

Give details of the individuals, organizations, groups or other legal entities who take responsibility for initiating, managing, sponsoring and/or financing the review. Any unique identification numbers assigned to the review by the individuals or bodies listed should be included.

None

13 Conflicts of interest

List any conditions that could lead to actual or perceived undue influence on judgements concerning the main topic investigated in the review.

Are there any actual or potential conflicts of interest?

None known

14 Collaborators

Give the name, affiliation and role of any individuals or organisations who are working on the review but who are not listed as review team members.

Title	First name	Last name	Organisation details

Review methods

15 Review question(s)

State the question(s) to be addressed / review objectives. Please complete a separate box for each question.

We are going to conduct a Bayesian network analysis to evaluate the relative effect of different renin angiotensin system blockades methods, including angiotensin receptor-blockers(ARB), angiotensin-converting enzyme inhibitor(ACEI) and ACEI+ARB in patients with IgA nephropathy.

16 Searches

Give details of the sources to be searched, and any restrictions (e.g. language or publication period). The full search strategy is not required, but may be supplied as a link or attachment.

PRISMA (PRISMA for Network Meta-Analyses) guidelines would be used in this study. We would search PubMed, the Cochrane Liberary, Embase ,CBMdisc, Wanfang database, CNKI with the PICOS strategy by advanced researches and Mesh researches with cut-off date of October 2017 in English and Chinese. The text words for searching include “IgA nephropathy, proteinuria, albuminuria, microalbuminuria, angiotensin receptor-blockers, ARB, angiotensin-converting enzyme inhibitor, ACEI, the names of currently available ARBs or ACEI (losartan, valsartan, irbesartan, candesartan, telmisartan, eprosartan, olmesartan, imidapril, enalapril, lisinopril, captopril, cilazapril, ramipril, perindopril, and fosinopril)”. Meanwhile,we will search for additional studies in the reference lists of all identified publications, including relevant meta-analyses and systematic reviews.

17 URL to search strategy

If you have one, give the link to your search strategy here. Alternatively you can e-mail this to PROSPERO and we will store and link to it.

18 Condition or domain being studied

Give a short description of the disease, condition or healthcare domain being studied. This could include health and wellbeing outcomes.

Chronic kidney disease(CKD) has become a significant public health problem. National Center for Chronic Disease Prevention and Health Promotion reported a 15% overall prevalence with CKD among adults in United States,suggesting that the population with CKD in 2017 has reached 30 million. IgA nephropathy has been the common CKD, accounting for 20%-40% of primary glomerulopathy in Asia and 10%-20% in west Europe. Among the patients with IgA nephropathy, about 1/3 of them would deteriorate to end stage renal disease(ESRD) in the coming decade and 5% of them would have a rapid deterioration in renal function in a short time resulting in an acute kidney injury. Proteinuria is one of the frequent symptoms in IgA nephropathy, which also has been perceived as a strong marker of kidney damage in IgA nephropathy, relating with an ascending risk of CKD progression.The patient who have a lot of proteinuia will get a poor prognosis. Currently, the common treatments for IgA nephropathy include fish oils, anticoagulants, antihypertensive agents, surgical tonsillectomy,renin angiotensin system blockades and immunosuppressive agents. Though there is lacking consensus in treatment protocols due to different clinical and pathological manifestations of IgA nephropathy, KIDGO(Kidney disease improving global outcomes) Guideline in 2012 pointed out the importance of renin angiotensin system blockades,including angiotensin converting-enzyme inhibitor (ACEI) and angiotensin-II receptor blocker (ARB), in the treatment of proteinuria in IgA nephropathy, which

would help protecting renal function by reducing proteinuria. Previous clinical studies and meta-analysis have testified that patients with IgA nephropathy can get a reduction of proteinuria by the treatment of ACEI / ARB alone or a combination of ACEI with ARBs. However, it remains unclear which therapeutic strategy may have better therapeutic effect on patients with IgA nephropathy in terms of a reduction of proteinuria and protecting renal function. Thus,we are going to conduct a Bayesian network analysis to evaluate the relative effect of these three therapeutic strategies in patients with IgA nephropathy.

19 Participants/population

Give summary criteria for the participants or populations being studied by the review. The preferred format includes details of both inclusion and exclusion criteria.

patients with IgA nephropathy

20 Intervention(s), exposure(s)

Give full and clear descriptions of the nature of the interventions or the exposures to be reviewed

angiotensinreceptor-blockers, ARB, angiotensin-converting enzyme inhibitor, ACEI, the names of currently available ARBs or ACEI (losartan, valsartan, irbesartan, candesartan, telmisartan, eprosartan, olmesartan, imidapril, enalapril, lisinopril, captopril, cilazapril, ramipril, perindopril, and fosinopril),

21 Comparator(s)/control

Where relevant, give details of the alternatives against which the main subject/topic of the review will be compared (e.g. another intervention or a non-exposed control group).

angiotensinreceptor-blockers, ARB, angiotensin-converting enzyme inhibitor, ACEI,placebo,other Antihypertensive Agents

22 Types of study to be included

Give details of the study designs to be included in the review. If there are no restrictions on the types of study design eligible for inclusion, this should be stated.

Randomized controlled trials(RCTs)

23 Context

Give summary details of the setting and other relevant characteristics which help define the inclusion or exclusion criteria.

24 Primary outcome(s)

Give the most important outcomes.

urinary protein excretion:Urinary total proteinuria/Urinary albumin excretion rate

Give information on timing and effect measures, as appropriate.

25 Secondary outcomes

List any additional outcomes that will be addressed. If there are no secondary outcomes enter None.

Serum creatinine/Glomerular filtration rate. blood pressure

Give information on timing and effect measures, as appropriate.

26 Data extraction (selection and coding)

Give the procedure for selecting studies for the review and extracting data, including the number of researchers involved and how discrepancies will be resolved. List the data to be extracted.

Data will be extracted from all primary studies including article information (Jadad score,first author's name ,publication year, regions), participants characteristics (patient characteristics,sample size, mean age, sex),intervention (specific medications used, duration of treatment, and number of dropouts) ,outcomes (urinary total proteinuria ,urinary albumin excretion rate, serum creatinine, glomerular filtration rate ,changes of systolic blood pressure (SBP), diastolic blood pressure (DBP))by two individuals independently. And we will solve the disagreements by discussion with a third investigator to reach consensus.

27 Risk of bias (quality) assessment

State whether and how risk of bias will be assessed, how the quality of individual studies will be assessed, and whether and how this will influence the planned synthesis.

We will use the Jadad scale to assess the methodological quality of studies,which mainly evaluated three aspects(Randomisation, Blinding, Withdrawals and dropouts) of all the studies. As a matter of fact, it is a five-point quality scale. Scoring =2 points was defined as low-quality, while scoring=3 points was ranked as high-quality studies. Meanwhile ,we will access the consistency and inconsistency with Bayesian method to explore the discrepancy of all studies and difference between direct and indirect comparisons. What's more, contribution figure and comparison-adjusted funnel plot will also be conducted to show the publication bias of this analysis.

28 Strategy for data synthesis

Give the planned general approach to be used, for example whether the data to be used will be aggregate or at the level of individual participants, and whether a quantitative or narrative (descriptive) synthesis is planned. Where appropriate a brief outline of analytic approach should be given.

The pair-wise meta-analysis of the same interventions, the basic network diagram and the Bayesian network analysis will be conducted by using ADDIS 1.16.5 soft[12] with consistency random-effect models.Statistical heterogeneity,consistency and inconsistency ,contribution and publication bias will be done with Stata 14.0.

29 **Analysis of subgroups or subsets**
Give any planned exploration of subgroups or subsets within the review. 'None planned' is a valid response if no subgroup analyses are planned.
Urinary total proteinuria >1.0g or Urinary total proteinuria<1.0g

Review general information

30 **Type and method of review**
Select the type of review and the review method from the drop down list.
Network meta-analysis
Complementary therapies

31 **Language**
Select the language(s) in which the review is being written and will be made available, from the drop down list. Use the control key to select more than one language.
English
Will a summary/abstract be made available in English?
Yes

32 **Country**
Select the country in which the review is being carried out from the drop down list. For multi-national collaborations select all the countries involved. Use the control key to select more than one country.
China

33 **Other registration details**
Give the name of any organisation where the systematic review title or protocol is registered together with any unique identification number assigned. If extracted data will be stored and made available through a repository such as the Systematic Review Data Repository (SRDR), details and a link should be included here.

34 **Reference and/or URL for published protocol**
Give the citation for the published protocol, if there is one.
Give the link to the published protocol, if there is one. This may be to an external site or to a protocol deposited with CRD in pdf format.

35 **Dissemination plans**
Give brief details of plans for communicating essential messages from the review to the appropriate audiences.
Do you intend to publish the review on completion?

36 **Keywords**
Give words or phrases that best describe the review. (One word per box, create a new box for each term)

37 **Details of any existing review of the same topic by the same authors**
Give details of earlier versions of the systematic review if an update of an existing review is being registered, including full bibliographic reference if possible.

38 **Current review status**
Review status should be updated when the review is completed and when it is published.
Ongoing

39 **Any additional information**
Provide any further information the review team consider relevant to the registration of the review.

40 **Details of final report/publication(s)**
This field should be left empty until details of the completed review are available.
Give the full citation for the final report or publication of the systematic review.
Give the URL where available.
