# Supplementary material for: Comparative efficacy of different renin angiotensin system blockade therapies in patients with IgA nephropathy: a Bayesian network meta-analysis of 17 RCTs
Source: PeerJ. 2021 Jul 6;9:e11661. doi: 10.7717/peerj.11661 (PMC8269645; doi:10.7717/peerj.11661)
Supplement: Supplemental Information 5 — This document explains the position of network meta-analysis. Bayesian network analysis is an extension of traditional meta-analysis. It has allowed the estimation of metrics for all possible comparisons in the same model, simultaneously gathering direct and indirect evidence, while traditional systematic reviews and pairwise meta-analyses usually compares only two interventions at the time. How to choose suitable therapeutic strategies is a common clinical problem, but no clinical studies have focused on all of these therapies. Thus, here, we tried to offer a reference to clinical practitioners. [file peerj-09-11661-s005.pdf]

https://doi.org/10.18549/PharmPract.2017.01.943

Review

Network meta-analysis: a technique to gather evidence from direct and indirect comparisons

Fernanda S. TONIN, Inajara ROTTA, Antonio M. MENDES, Roberto PONTAROLO.
Received (first version): 13-Feb-2017 Accepted: 7-Mar-2017

Abstract

Systematic reviews and pairwise meta-analyses of randomized controlled trials, at the intersection of clinical medicine, epidemiology and statistics, are positioned at the top of evidence-based practice hierarchy. These are important tools to base drugs approval, clinical protocols and guidelines formulation and for decision-making. However, this traditional technique only partially yield information that clinicians, patients and policy-makers need to make informed decisions, since it usually compares only two interventions at the time. In the market, regardless the clinical condition under evaluation, usually many interventions are available and few of them have been studied in head-to-head studies. This scenario precludes conclusions to be drawn from comparisons of all interventions profile (e.g. efficacy and safety). The recent development and introduction of a new technique – usually referred as network meta-analysis, indirect meta-analysis, multiple or mixed treatment comparisons – has allowed the estimation of metrics for all possible comparisons in the same model, simultaneously gathering direct and indirect evidence. Over the last years this statistical tool has matured as technique with models available for all types of raw data, producing different pooled effect measures, using both Frequentist and Bayesian frameworks, with different software packages. However, the conduction, report and interpretation of network meta-analysis still poses multiple challenges that should be carefully considered, especially because this technique inherits all assumptions from pairwise meta-analysis but with increased complexity. Thus, we aim to provide a basic explanation of network meta-analysis conduction, highlighting its risks and benefits for evidence-based practice, including information on statistical methods evolution, assumptions and steps for performing the analysis.

Keywords

Network Meta-Analysis; Evidence-Based Practice; Treatment Outcome; Decision Support Techniques

INTRODUCTION

In the last decade, network meta-analysis (NMA) and multiple treatment comparisons (MTC) of randomized controlled trials (RCT) has been introduced as an extension of pairwise meta-analysis, with the advantage to facilitates indirect comparisons of multiple interventions that have not been studied in head-to-head studies.[1,2] These new methods are attractive for clinical researchers because they seem to respond to their main concern: determining the best available intervention. Moreover, national agencies for health technology assessment and drug regulators increasingly use such methods.[3,4] However, although assumptions underlying pairwise meta-analyses are well understood, those concerning NMA are perceived to be more complex and prone to misinterpretation.[5,6] Compared with pairwise meta-analyses, network meta-analyses allow the visualisation of a larger amount of evidence, estimation of the relative effectiveness among all interventions, and rank ordering of the interventions.[5,7]

The conduction of NMA still poses multiple challenges that should be carefully considered when utilizing such methods. Thus, we aim to describe the underlying assumptions and methods used in indirect comparisons and network meta-analyses, as well as to explain results interpretation, and characterize this statistical tool as an essential piece of evidence-based practice.

Fernanda S. TONIN. MSc. (Pharm). Pharmaceutical Sciences Postgraduate Programme, Federal University of Paraná. Curitiba (Brazil). stumpf.tonin@ufpr.br
Inajara ROTTA. PhD. Pharmacy Service, Hospital de Clínicas, Federal University of Paraná. Curitiba (Brazil). inarotta@gmail.com
Antonio M. MENDES. MSc. (Pharm). Pharmaceutical Sciences Postgraduate Programme, Federal University of Paraná. Curitiba (Brazil). mmendesantonio@gmail.com
Roberto PONTAROLO. PhD. Department of Pharmacy, Federal University of Paraná. Curitiba (Brazil). pontarolo@ufpr.br

Meta-analyses and clinical practice

Systematic reviews and meta-analyses of RCT, being at the intersection of clinical medicine, epidemiology, and statistics, are positioned at the top of evidence-based hierarchy and are important tools for drug approval, clinical protocol formulation and decision making.[8,9] Although meta-analysis has been employed in clinical practice since the 1980s and its use became widespread in the 1990s, possibly due to the establishment of the Cochrane Collaboration, the methods to refine, reduce bias, and especially improve statistical analyses have developed slowly.[10-12]

Traditional meta-analytical methods refer to pairwise comparisons between an intervention and a control, typically a placebo or other active intervention.[13,14] This standardized approach allows examining the existing literature on a specific issue to determine whether a conclusion can be reached regarding the effect of a treatment. If it is well conducted, the strength of meta-analysis lies in its ability to combine the results from various small studies that may have been underpowered to detect a statistically significant difference between one intervention and another (Figure 1).[12,15,16] However, this traditional technique only partially yields information that clinicians, patients and policy-makers need to make informed decisions on prevention, diagnosis, and treatments, since usually more than two health

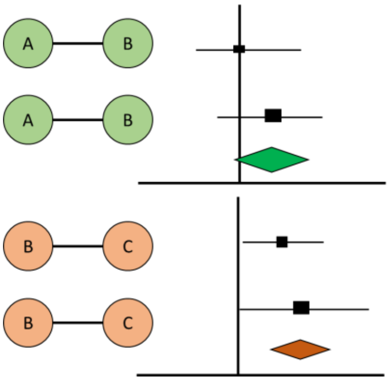

Figure 1. Example of pairwise meta-analyses.
In the literature we can found RCT directly comparing interventions (e.g. A versus B in green; and B versus C in orange). Each RCT produce an effect in the meta-analyses (e.g. odds ratio, risk ratio, mean difference) represented by the lines in the graph and a global effect measure (diamond) that represents the reunion of the effects of the included studies. However, in this model is not possible to compare interventions A and C.

technologies are available in the market for certain conditions.[16-19] Nonetheless, there is often a lack of, or limited, evidence in the literature from head-to-head clinical trials, which hampers conclusions being drawn from comparisons of drug efficacy and safety profiles. This situation occurs partly due to commercial interests and countries' regulatory approval processes, where placebo-controlled trials are normally sufficient for demonstration of the efficacy of a new drug. In addition, carrying out an RCT with active comparators demands large sample sizes, being an expensive undertaking.[20-22]

Given this unsettled scenario, recent statistical advances have resulted in the development of methods that allow the estimation of efficacy/safety metrics for all possible comparisons in the same model, regardless of whether there have been direct, head-to-head comparisons in clinical trials.[6,17,23] This is important, because costs involved in the development of new or unnecessary clinical studies may be reduced. Moreover, these analyses may offer a first overview of the entire set of a clinical condition (e.g. available treatments, existing comparisons, risks and benefits of each therapeutic option) and guide the conduct of new researches (e.g. clinical trials and observational studies).

The evolution of indirect meta-analytical methods

The introduction of the adjusted indirect treatment comparison (ITC) method – also called anchored ITC, first proposed by Bucher *et al*. (1997) – has provided an initial solution accounting for treatments that have not been directly compared in literature.[24] This model was developed with the odds ratio (OR) as the measure of treatment effect, and was specifically designed for the indirect comparison of A versus C when direct evidence of A versus B and B versus C was available. Thus, a global effect – similar to that generated by pairwise meta-analysis – is created for each comparison (A versus B, B versus C, and A versus C). However, this model has the limitation that it can only be applied to data generated from two-arm trials involving simple indirect comparison of three treatments (Figures 2 and 3).[25,26]

After that, Lumley[27] developed an indirect treatment comparison technique, known as network meta-analysis (NMA), to compare two treatments in the situation where an indirect comparison between two treatments of interest can be obtained through more than one common comparator or linking treatment. For instance, consider a setting where there is interest in performing an indirect comparison between treatment A and treatment B. If trials have separately compared treatment A to C, treatment B to C, treatment A to D, and treatment B to D, Lumley's

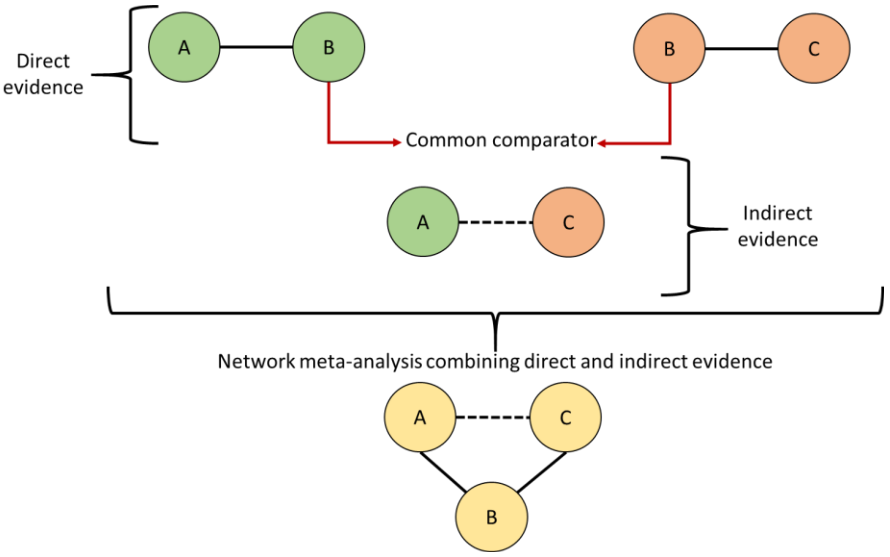

Figure 2. Direct and indirect evidence.
In the literature we can found RCT directly comparing interventions (e.g. A versus B in green; and B versus C in orange). Each circle represents an intervention and lines represent direct comparisons. Dashed lines are for indirect comparison. An global effect value is generated for each comparison (direct or indirect). Indirect evidence is generated by using B as common comparator for the comparison of A versus C (model proposed by Bucher). Network meta-analysis combining both direct and indirect evidence may be built.

BY NC ND

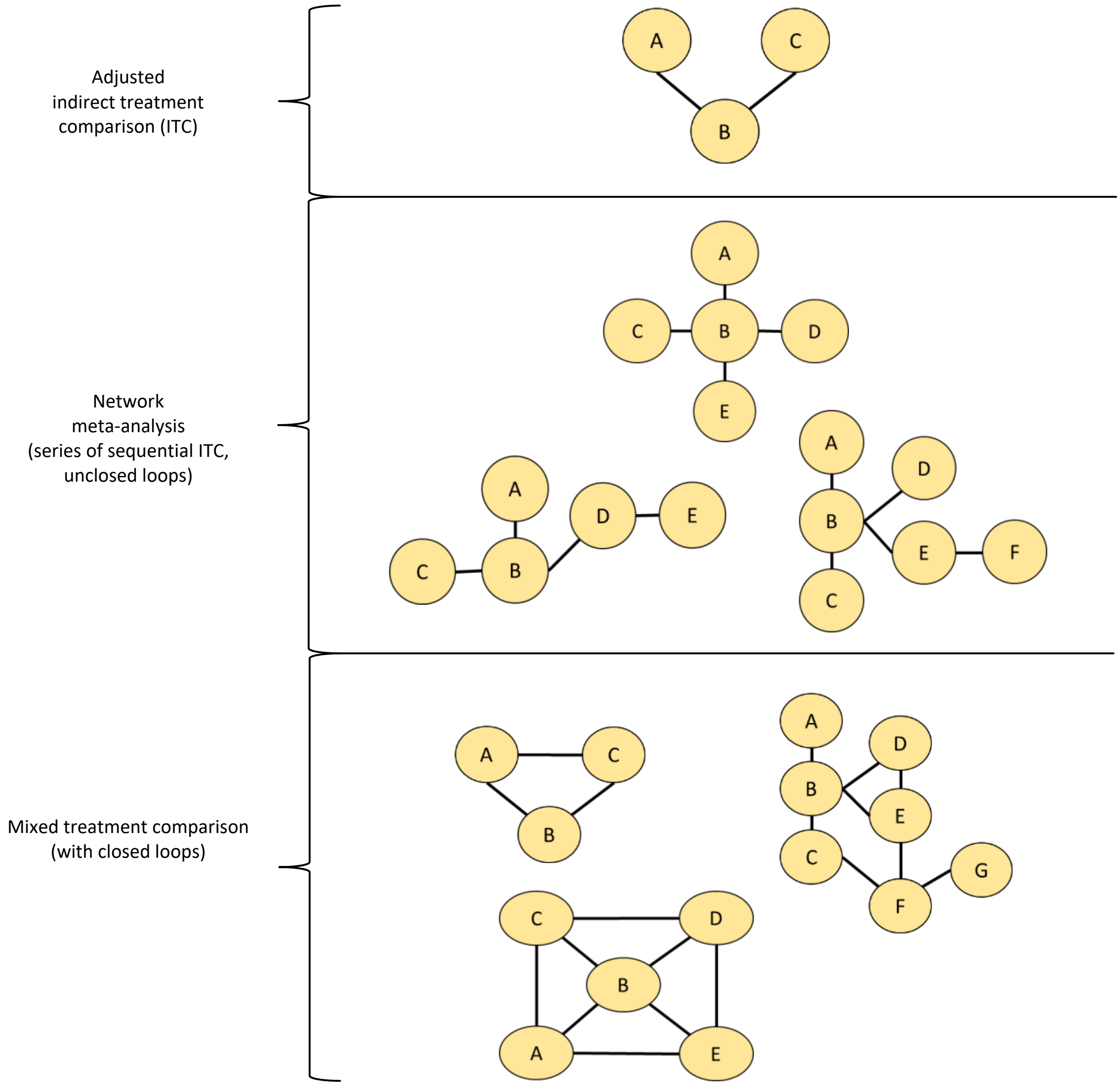

Figure 3. Network diagrams and definitions (Examples of networks geometries and evolution of statistical concepts). First panel: Adjusted Indirect Treatment Comparison (ITC) proposed by Bucher (simple indirect comparison); Second Panel: Network meta-analysis proposed by Lumley (open loops meta-analysis); Third Panel: Mixed Treatment Comparison proposed by Lu and Ades as an improvement of Network meta-analysis from Lumley. Together, these meta-analytical process are also called "network meta-analysis" and cover direct and indirect comparisons in the same model.

method allows investigators to incorporate results from trials in which the common comparator was C, as well as trials in which the common comparator was D. Thus, more than one common treatment can be used to conduct an indirect comparison between two treatments. NMA also allows determining the amount of agreement between the results obtained when different linking treatments are used. Lumley has indicated that if the indirect comparison between two treatments yields the same result, regardless of which common comparator is used (C or D), there is a greater likelihood that the indirect treatment comparison represents the true relationship between the interventions. On the other hand, if there is a discrepancy in the results, "incoherence" exists, and Lumley has provided mechanisms to measure this incoherence (also called "inconsistency" in the network). In this model, different from that proposed by Bucher, we may account for both direct and indirect evidence at the same time.

Finally, in order to provide an even more sophisticated method for quantitatively addressing both direct and indirect comparisons of several competing interventions, Lu and Ades[28] have improved NMA techniques and provided information on mixed/multiple treatment comparison meta-analysis (MTC or MTM) (Figure 3). Because of its similarity to the model proposed by Lumley, MTC has also been referred to as "network meta-analysis." Lu and Ades have described the statistical methods for performing MTC in a Bayesian framework with the aim of strengthening inference concerning the relative efficacy of two treatments by including both direct and indirect comparisons of these treatments.[23] They have also facilitated simultaneous inference regarding all treatments by potentially ranking these treatments. Calculations of the probability of one treatment being the best or worst for a specific outcome through rank orders or rankograms

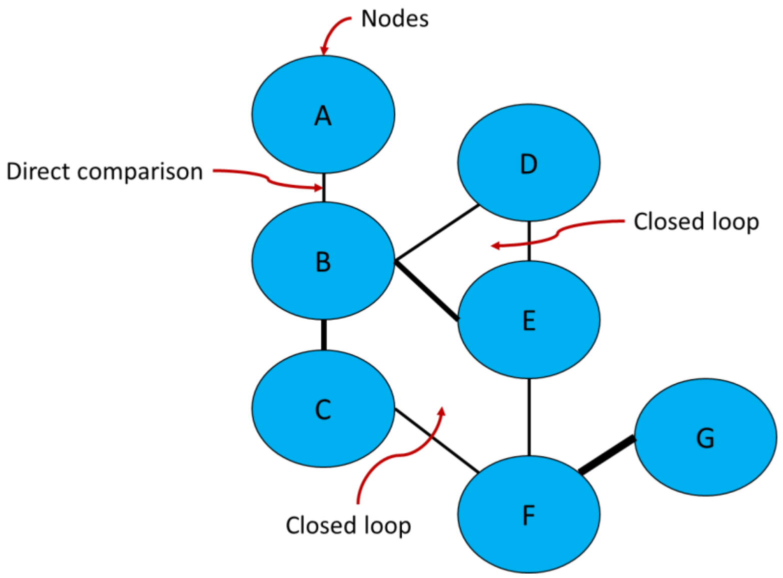

Figure 4. Network diagram – basic components.
A network is composed by at least three nodes (interventions or comparators) connected by lines (direct comparisons). In this diagram, lines width is proportional to the number of direct evidence available in the literature. Closed loops may be formed according to the availability of direct and indirect evidence on the literature (e.g. B vs. C vs. E vs. F represent a closed loop; B vs. D vs. E is another closed loop). Indirect evidence is calculated using a common comparator (e.g. estimations between A and D are made through B; estimations between E and G are made through F).

(graphical methods) are usually employed, and facilitate the interpretation of the results.[26,29]

Networks of any kind (ITC, NMA or MTC) may take on different shapes and are usually visually represented by figures, called network plots or graphs (Figure 4). The nodes (circles) usually represent the interventions or technologies under evaluation. The lines that connect the interventions represent the direct comparisons available in the literature. The comparisons that may be built between two interventions from those direct evidences are called indirect comparisons. The set of direct and indirect statistical comparisons is the NMA. Some networks can be created accounting for the number of direct evidences available in the literature (line width) and/or the volume of studies referring to each intervention (size of nodes). Poorly connected networks depend extensively on indirect comparisons. Meta-analyses of such networks may be less reliable than those from networks where most treatments have been compared against each other. Qualitative description of network geometry should be provided and

Table 1. Concepts and definitions in the field of network meta-analysis

**Common comparator**	is the anchor to which treatment comparisons are anchored. If a network has three treatments (A, B and C) and A is directly linked to B while C is also directly linked to B; the common comparator of this network is B.
**Direct treatment comparison**	comparison of two interventions through studies that directly compare active drugs (head-to-head trials) or comparison with placebo
**Adjusted Indirect treatment comparison (ITC)**	is estimate using separate comparisons of two interventions (e.g. A versus B; B versus C) and takes into account a common comparator (in this case, B). Thus, the direct treatment effects of each intervention against the common comparator are used to estimate an indirect evidence between the two interventions (Bucher ITC analyses)
**Network meta-analysis (NMA) or Mixed treatment comparison/meta-analyses (MTC or MTM)**	these terms, which are often used interchangeably, refer to situations involving the simultaneous comparison of three or more interventions. Any NMA treatments consisting of strictly unclosed loops can be thought of as a series of ITCs. In MTC, both direct and indirect information is available to inform the effect size estimates for at least some of the comparisons; visually, this is shown by closed loops in a network graph. Closed loops are not required to be present for every comparison under study. "Network meta-analysis" is an inclusive term that incorporates the scenarios of both indirect and mixed treatment comparisons.
**Network diagram and geometry**	basis of this network analysis is a network diagram (graph) where each node represents an intervention and the connecting lines between them represent one or more RCTs in which interventions have been directly compared. The description of characteristics of the network of interventions, which may include use of numerical summary statistics is considered a network geometry evaluation.
**Closed loop**	in the network diagram each comparison has both direct and indirect evidence. For example, the BC comparison has direct evidence from the BC trials and indirect evidence from the AB and AC trials (and similarly for the AB and AC comparisons).
**Rank order or rankeogram**	calculations of the probability of one treatment being the best, second best and so on for a specific outcome.
**Inconsistency or incoherence**	statistical conflicts in the network model (regarding source of evidence, degree of similarity of data, lack of consistent information) that should be investigated to guarantee the robustness of the model.

CC BY NC ND

https://doi.org/10.18549/PharmPract.2017.01.943

accompanied by a network graph or diagram for better understanding and interpretation of results.[30,31] For instance, closed loop refers to a part in the network where all the interventions are directly connected forming a closed geometry (e.g. triangle, square). In this case, both direct and indirect evidence exists. On the other hand, open or unclosed loops are referred to as incomplete connections in the network (loose ends). Some of the most common terms and definitions of NMA are shown in Table 1.

Prior to 2008, very few systematic reviews containing NMAs were published. However, there has been a marked growth of its use for the evaluation of health technologies and procedures (e.g. surgeries, transplants, psychological therapies), especially pharmacological interventions. To date, more than 360 NMAs published by more than 30 different countries have been recorded in the scientific literature on drug interventions. The most evaluated clinical conditions are cardiovascular diseases, oncological disorders, mental health disorders and infectious diseases. There are also around 100 published articles describing statistical strategies, alternative methods and providing software or algorithms to conduct NMA.

NMA assumptions

NMA (covering all types of statistical analyses shown above) has matured over the last few years and models are available for all types of underlying data and summary effect measures, being implemented in both Frequentist and Bayesian frameworks with different software.[13,31-36]

The key feature of NMA is that it allows the synthesis of direct and indirect estimates for the relative effects of many competing treatments for the same health condition. Diversity and strength of a network is determined by the number of different interventions and comparisons that are available, how represented they are in network and the evidence they carry.[17,29] However, NMA inherits all challenges present in a standard pairwise meta-analysis, but with increased complexity due to the multitude of comparisons involved (heterogeneity, consistency, precision), which may generate inconsistency or incoherence in the model.

Inconsistency can arise from the studies' characteristics – since they are usually differently designed, or when both direct and indirect estimates of an effect size are available in the literature but are divergent (e.g. A-C is measured both directly and via B as an indirect estimate). Examples of causes of inconsistency:

- Participants in head-to-head trials of A-B are different from those in B-C and A-C studies.
- Versions of treatment B are different in studies of A-B and studies of B-C (e.g. doses, regimen, type of treatment, etc.).
- Studies of different comparisons were undertaken in different periods, different settings or contexts.

To deal with these issues, NMA adopts some assumptions that should be followed to design the study: (i) similarity or exchangeability, (ii) homogeneity and (iii) transitivity or consistency. The first two assumptions also apply to pairwise meta-analyses.

- Similarity assumption: the selection of trials to compose the NMA should be based on rigorous criteria and thus studies should be similar. Besides study population, design, and outcome measures, trials must be comparable on effect modifiers to obtain an unbiased pooled estimate. Effect modifiers are study and patient characteristics (e.g. age, disease severity, duration of follow-up...) that are known to influence treatment effect of interventions. Imbalanced distribution of effect modifiers between studies can bias comparisons, resulting in heterogeneity and inconsistency. That is, when similar, all studies measure the same underlying relative treatment effects, and any observed differences are only due to chance. For instance, studies comparing A versus B should be similar to those comparing B versus C.[32,37,38]
- Homogeneity assumption: there must be no relevant heterogeneity between trial results in pairwise comparisons.[32,37,38]
- Consistency and transitivity assumptions: there must be no relevant discrepancy or inconsistency between direct and indirect evidence. It means that the desirable relationship between direct and indirect sources of evidence for a single comparison is typically expressed in terms of consistency (comparability). The statistical manifestation of consistency is called transitivity. For instance, in closed loop networks both direct and indirect evidence are available and it is assumed that for each pairwise comparison (A-B; B-C and A-C), direct and indirect estimates should be consistent. Violation of these assumptions transgresses the theory of transitivity, where one cannot conclude that C is better than A from trial results that have already proven that C is better than B and B is better than A.[26,35]

Thus, when planning a network meta-analysis, it is important to assess the effect modifiers and include traits such as average patient age, gender distribution, disease severity, and a wide range of other plausible features. For NMA to produce valid results, it is important that the distribution of effect modifiers is similar, since this balance increases the plausibility of reliable findings from an indirect comparison. Authors should present systematic (and even tabulated) information regarding these characteristics whenever available. This information helps readers to empirically evaluate the validity of the assumption of transitivity by reviewing the distribution of potential effect modifiers across trials.[18,30,39]

Statistical methods in NMA

Analysis of network involves pooling of individual study results. As already mentioned, factors such as total number of trials in a network, number of trials with more than two comparison arms, heterogeneity (i.e., clinical, methodological, and statistical variability within direct and indirect comparisons), inconsistency, and bias may influence effect estimates obtained from NMA.[6,17,23]

NMA can be performed within either a frequentist or a Bayesian framework. Frequentist and Bayesian approaches

A	1.12 (0.31 - 3.44)	1.98 (0.22 – 2.25)	**<u>1.72 (1.05 - 2.91)</u>**	**<u>2.88 (1.27 - 3.04)</u>**
	B	**<u>0.45 (0.15 – 0.92)</u>**	1.07 (0.30 - 1.39)	**<u>1.60 (1.02 – 3.87)</u>**
		C	1.26 (0.74 - 2.14)	**<u>2.81 (1.23 - 2.73)</u>**
			D	**<u>1.94 (1.25 - 3.63)</u>**
				Placebo

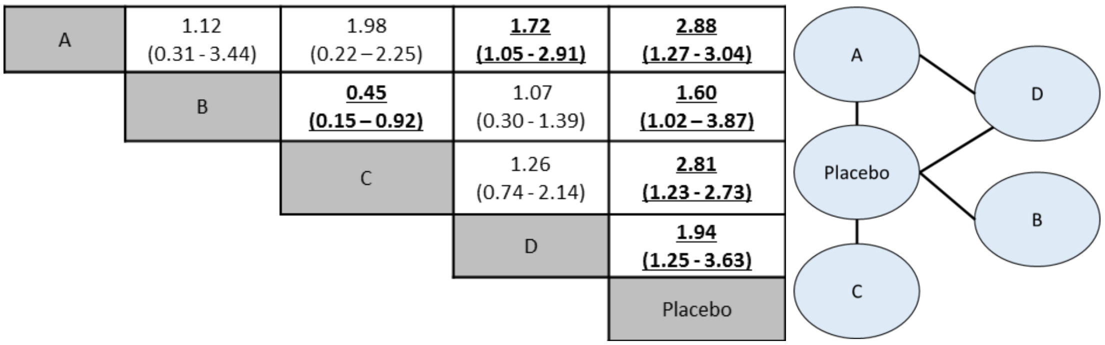

Figure 5. Tables with results of MTC analyses: pooled effect sizes for the outcomes of efficacy (e.g. cure of a disease). On the right, the network plot shows four interventions and a placebo. Intervention D and placebo act as common comparators. On the left, in the consistency table, drugs are reported alphabetically. Comparisons between treatments should be read from left to right (i.e. treatment 1 versus treatment 2). The estimate effect measure (e.g. odds ratio – OR followed by 95% CrI) is in the cell in common between the row-defining treatment and column-defining treatment. Values of OR higher than 1 favour the occurrence of the outcome in the defined-treatment 1. Values of OR lower than 1 favour the outcome to the defined-treatment 2. Significant results are in bold and underlined. For instance, A versus B value is 1.12 (0.31-3.44) and interventions have no significant differences. A versus D value is 1.72 (1.05-2.91), favouring intervention A as most effective.

to statistics differ in their definitions of probability. Frequentist analyses calculate the probability that the observed data would have occurred under their sampling distribution for hypothesized values of the parameters. The results of the analysis are given as a point estimate (effect measures such as odds ratio - OR, risk ratio – RR, mean difference) with a 95% confidence interval (CI), similar to pairwise meta-analysis results.[40,41]

Bayesian analyses rely on the probability distribution of all the model parameters given the observed data, and additionally prior beliefs (e.g. from external information) about the values of the parameters. They fully cover the uncertainty in the parameters of interest and thus can make direct probability statements about these parameters (e.g., the probability that one intervention is superior to another).[20,42] Results are usually presented as a point estimate with a 95% credibility interval (ICr) and are performed with Markov Chain Monte Carlo (MCMC) simulations, which allows reproduction of the model several times until convergence. One advantage of the Bayesian approach is that it has a straightforward way of making predictions, and includes the possibility of incorporating different sources of uncertainty, with a more flexible statistical model.[43,44]

The presentation of results is usually made for the direct evidence (all possible pairwise meta-analyses), indirect evidence and combined evidence. The combined evidence is often represented in tables of consistency. Usually, results are shown as the value of the effect measure (OR, RR, mean difference) and IC or ICr. An example of result presentation is shown in Figure 5. As we can see in this figure, the network has four interventions (A, B, C and D) and placebo as components. Both direct and indirect comparisons were performed and these results are given for the mixed treatment comparison. The interpretation of the results is similar to those from pairwise meta-analysis and is given by pairs of comparisons (e.g. A vs. B; A vs. C...). As we can see in Figure 5, all interventions were better than placebo for the evaluated outcome (e.g. efficacy). Intervention A was also better than D while C was more favourable than B. This information can guide decision making about these available therapeutic options for a clinical condition in a health set. They account for all comparisons at the same time even if there are no head-to-head trials (direct evidence) in the literature.

Similar to traditional pairwise meta-analysis, NMA can utilize the fixed effect or the random effect approach. Fixed effect approach assumes that all studies are trying to assume one true effect size and any difference between estimates from different studies is attributable to sampling error only (within study variation). A random effects approach assumes that in addition to sampling error, observed difference in effect size considers the variation of true effect size across studies (between study variation), otherwise called heterogeneity, attributed to severity of disease, age of patients, dose of drugs, follow-up period, among others. Extending this concept to NMA, it is expected that effect size estimates not only vary across studies but also across comparisons (direct and indirect). Both models should be tested for each network.[45,46]

Different methods to evaluate potential differences in relative treatment effects estimated by direct and indirect comparisons are grouped as local approaches and global approaches:

- Local approaches (e.g., node-splitting method) assess the presence of inconsistency for a particular comparison in the network, comparing the results for robustness. Node-splitting analysis is an alternative method to evaluate inconsistency, since it assesses whether direct and indirect evidence on a specific node (the split node of a closed loop in the network) are in agreement.[34,47]
- The global approaches consider the potential for inconsistency in the network as a whole. Statistical heterogeneity can be checked by Cochran's Q test and quantified by I2 statistics.[23,30,47]

Considering the Bayesian approach, besides model convergence (e.g. shown by MCMC simulations), it is also important to choose an NMA model that better fits the included data. For this, effect sizes estimates, changes in heterogeneity and statistical methods such as DIC (deviance information criteria) should be used for model fitting.[30,43,48]

Ranking	Efficacy of treatments (e.g. cure)				
	A	B	C	D	Placebo
1	0.46	0.15	0.20	0.18	0.01
2	0.42	0.07	0.48	0.03	0.00
3	0.10	0.25	0.21	0.44	0.00
4	0.01	0.49	0.08	0.28	0.14
5	0.01	0.04	0.03	0.07	0.85

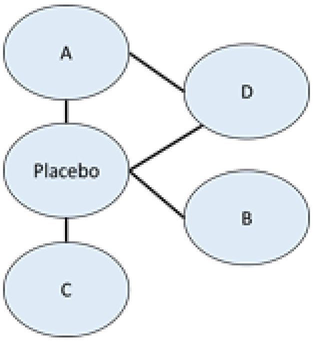

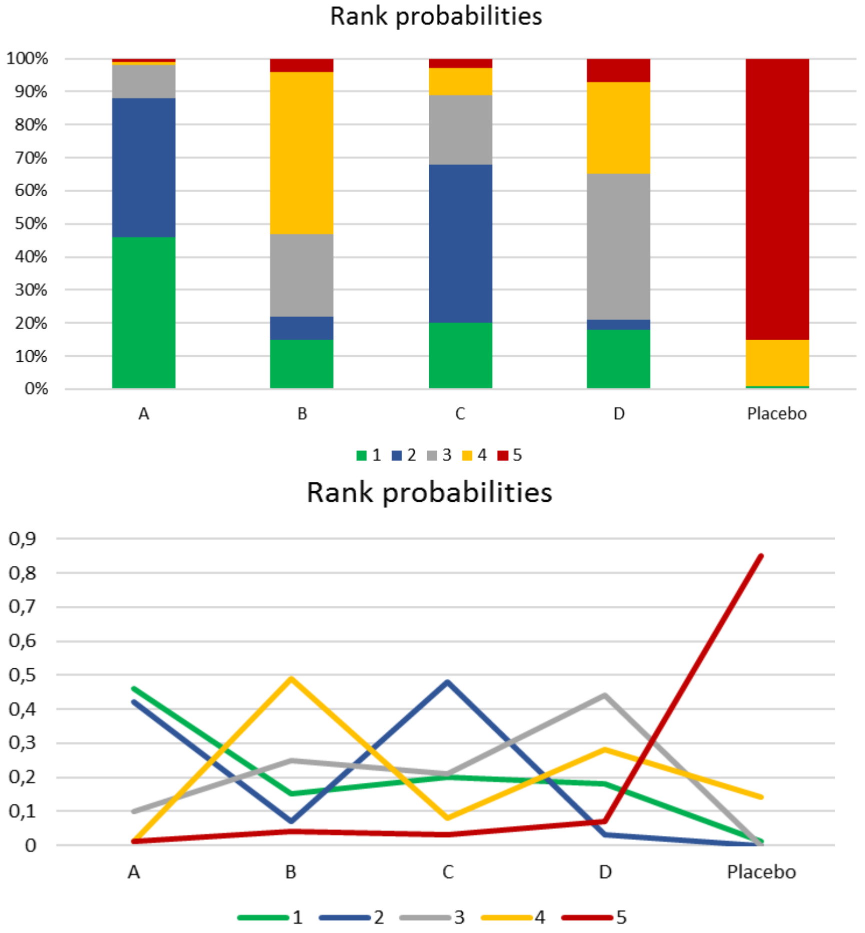

Figure 6. Rank probabilities representations.
For a network with five nodes (A, B, C, D and placebo), after the interpretation of the model of consistency (figure 5), we can order the intervention using different tools (graphics, tables). In each one of them, the probabilities of each intervention to be the best (1st in the rank), second best, third, fourth and last in the rank (5th position - worst therapy) are calculated. First panel: probabilities are given such as percentages. Rank probabilities sum to one, both within a rank over treatments (horizontal) and within a treatment over ranks (columns). Intervention A has 46% (0.46) of probability of being the best drug (first in the rank), followed by C (48%), D (44%), B (49%) and placebo (85%). This same scenario is presented in the second panel and third panels (as a graphic illustration) where each intervention has a probability to be part of the first, second, third, fourth, fifth positions.

Another advantage of MTC analyses usually related to Bayesian method is the ability to provide treatment ranking probabilities (rank order or rankograms). It refers to the probabilities estimated for each treatment in a network for achieving a particular placement in an ordering of treatment effects from best to worst. That is, the chance for each intervention of being ranked as first, second, third, fourth and so on.[49,50] Rankings can be reported along with the corresponding estimates of comparisons between interventions (e.g. tables of consistency, results of meta-analyses). Rankings should be reported with probability estimates to minimize misinterpretation from focusing too much on the most likely rank. Several techniques are feasible to summarize relative rankings, and include graphical tools as well as different approaches for estimating ranking probabilities (Figure 6). Robust reporting of rankings may also include specifying statistics such as median ranks with uncertainty intervals, cumulative probability curves, and the surface under the cumulative ranking (SUCRA) curve.[19,36,51]

Table 2. Performing NMA: basic steps	
1. Define the review question and inclusion criteria	Similar to pairwise meta-analysis, the definition of the study question is important. Treatments of the network (nodes) should be precisely defined. Whenever possible all available drugs or treatments should be included in the NMA. Follow PRISMA-NMA extension guide and recommendations (e.g. Cochrane Collaboration) to conduct the systematic review and NMA.
2. Search and select studies	Ensure that the search is broad enough and all studies of interest are included.
3. Perform titles/abstract and full-text reading	These steps also should be performed systematically and carefully, since information on potential effect modifiers may violate the assumption of NMA transitivity.
4. Risk of bias assessment	All trials should be evaluated using methodological quality and risk of bias in order also to preserve similarity and consistency.
5. Extraction of data, network building and statistical analyses	Qualitative and quantitative data should be extracted from the included studies.<br>A first network draft can be drawn and its geometry should be evaluated.<br>Conduct the pairwise meta-analysis, build models for NMA using appropriate statistical methods and evaluate inconsistency.<br>Provide data on convergence and model fit.<br>Rank order analysis can also be provided.
6. Synthesis of results	Summarize results using appropriate approaches such as tables, diagrams, rankograms.
7. Interpretation of results and conclusions	Interpret the results in the context of the disease/clinical condition and available treatments.<br>Carefully interpret data, especially figures such as rankograms.<br>GRADE (Grading of Recommendations, Assessment, Development and Evaluation) approach and R-AMSTAR (Revised version of Assessing the Methodological Quality of Systematic Reviews tool) tools may be applied to evaluate the quality of the already published systematic reviews with NMA and the level of evidence of results.

Since NMAs are usually based on Bayesian statistics, robust softwares with well-designed program codes are required. The use of NMAs has grown rapidly in recent years, and more complex models are becoming increasingly common.[34,36,40,51-56] The most common choices of software are:

- WinBUGS: a commercial source with a large body of codes published in the literature. However, they can be slow and difficult to use.
- OpenBUGS: an open source version of WinBUGS. Operates as a standalone program or can be called from other statistical software such as R and SAS.
- ADDIS (Aggregate Data Drug Information System): an open source proof-of-concept system for decision support system GeMTC GUI component for NMA. However, it is not that flexible.
- JAGS (Just Another Gibbs Sampler): open source program for Bayesian inference. Operated from the command line or R. The modelling language is similar to WinBUGS and OpenBUGS.
- R: open source software for statistics. It uses packages such as: GeMTC (specifically designed for fitting NMA models); MCMCpack (for fitting specific types of MCMC models); LaplacesDemon (flexible R package for MCMC). It usually requires WinBUGS, OpenBUGS or JAGS for operation. The recent launch of R Studio (a platform that operates with R) made it easier to program NMAs.
- Python: a general purpose open source programming language. It uses the PyMC module for Bayesian inference.
- STATA: a commercial general-purpose, command-line driven software for statistics, can be used for building NMA (e.g. mvmeta command).
- SAS: a commercial software package for statistics, can be used for NMA modeling.

Steps for performing NMA

Despite the benefits of NMA, there is still controversy among researchers about the validity of using indirect treatment comparisons (indirect evidence) for decision making. The use of such evidence is particularly challenged when direct treatment comparisons (direct evidence) are also available.[31,35,38] Although it is often acknowledged that having the most up-to-date evidence is critical to clinical practice, it is equally important that optimal analytical methods are used to appraise the evidence and thus provide optimal evaluation of all the competing interventions at the same time.[30,57] As already highlighted, NMA may support approval and decision-making when lacking sufficient, direct, head-to-head trials, being a cheap and accessible tool.

Important key aspects rely on the conduct and reports of NMA, which may ensure consistency, robustness and reproducibility of data. It is also important to consider that often NMAs are accompanied by a previous systematic review process which should be well-designed and properly reported to avoid errors in the statistical analyses. However, currently available literature regarding the reporting of NMA is sparse, and several deficiencies in the conduct and presentation of NMA are apparent.

Table 2 shows some basic steps to guide NMA practice. The international PRISMA statment (Preferred Reporting Items for Systematic Reviews and MetaAnalyses)[18,30] has recently proposed an extension, called PRISMA-NMA, that incorporates network meta-analyses and provides guidelines on how to analyze and report data. This statement was designed to improve the completeness of reporting of systematic reviews and NMA, and also highlights educational information related to key considerations in the practice and use of this technique. The target audience includes authors and readers of network meta-analyses, as well as journal editors and peer reviewers.

Epilogue

NMA in all its formats and statistical approaches can provide findings of fundamental importance for the development of guidelines and for evidence-based decisions in health care. It represents an important extension of traditional pairwise meta-analyses and provides a more complete overview of a health set. However, appropriate use of these methods requires strict assumptions and standardization. Transparent, reproducible and detailed documentation is required so that the published findings of NMA can be suitably evaluated.

CONFLICT OF INTEREST

None.

FUNDING

This work was supported by the Brazilian National Council of Scientific Research (CNPq) and the Coordination for the Improvement of Higher Education Personnel (CAPES).

References

1. Jansen JP, Naci H. Is network meta-analysis as valid as standard pairwise meta-analysis? It all depends on the distribution of effect modifiers. BMC Med. 2013;11:159. doi: 10.1186/1741-7015-11-159
2. Jansen JP, Trikalinos T, Cappelleri JC, Daw J, Andes S, Eldessouki R, Salanti G. Indirect treatment comparison/network meta-analysis study questionnaire to assess relevance and credibility to inform health care decision making: an ISPOR-AMCP-NPC Good Practice Task Force report. Value Health. 2014;17(2):157-173. doi: 10.1016/j.jval.2014.01.004
3. Bafeta A, Trinquart L, Seror R, Ravaud P. Analysis of the systematic reviews process in reports of network meta-analyses: methodological systematic review. BMJ. 2013;347:f3675. doi: 10.1136/bmj.f3675
4. Bafeta A, Trinquart L, Seror R, Ravaud P. Reporting of results from network meta-analyses: methodological systematic review. BMJ. 2014;348:g1741. doi: 10.1136/bmj.g1741
5. Caldwell DM, Dias S, Welton NJ. Extending treatment networks in health technology assessment: how far should we go? Value Health. 2015;18(5):673-681. doi: 10.1016/j.jval.2015.03.1792
6. Cipriani A, Higgins JP, Geddes JR, Salanti G. Conceptual and technical challenges in network meta-analysis. Ann Intern Med. 2013;159(2):130-137. doi: 10.7326/0003-4819-159-2-201307160-00008
7. Debray TP, Schuit E, Efthimiou O, Reitsma JB, Ioannidis JP, Salanti G, Moons KG, GetReal W. An overview of methods for network meta-analysis using individual participant data: when do benefits arise? S Stat Methods Med Res. 2016:962280216660741. doi: 10.1177/0962280216660741
8. Paul M, Leibovici L. Systematic review or meta-analysis? Their place in the evidence hierarchy. Clin Microbiol Infect. 2014;20(2):97-100. doi: 10.1111/1469-0691.12489
9. Leucht S, Kissling W, Davis JM. How to read and understand and use systematic reviews and meta-analyses. Acta Psychiatr Scand. 2009;119(6):443-450. doi: 10.1111/j.1600-0447.2009.01388.x
10. Mills EJ, Bansback N, Ghement I, Thorlund K, Kelly S, Puhan MA, Wright J. Multiple treatment comparison meta-analyses: a step forward into complexity. Clin Epidemiol. 2011;3:193-202. doi: 10.2147/CLEP.S16526
11. Sutton AJ, Higgins JP. Recent developments in meta-analysis. Stat Med. 2008;27(5):625-650.
12. Chalmers I. The Cochrane collaboration: preparing, maintaining, and disseminating systematic reviews of the effects of health care. Ann N Y Acad Sci. 1993;703:156-163.
13. Dias S, Sutton AJ, Ades AE, Welton NJ. Evidence synthesis for decision making 2: a generalized linear modeling framework for pairwise and network meta-analysis of randomized controlled trials. Med Decis Making. 2013;33(5):607-617. doi: 10.1177/0272989X12458724
14. Rouse B, Chaimani A, Li T. Network meta-analysis: an introduction for clinicians. Intern Emerg Med. 2017;12(1):103-111. doi: 10.1007/s11739-016-1583-7
15. Berlin JA, Cepeda MS. Some methodological points to consider when performing systematic reviews in comparative effectiveness research. Clin Trials. 2012;9(1):27-34. doi: 10.1177/1740774511427062
16. Garg AX, Hackam D, Tonelli M. Systematic review and meta-analysis: when one study is just not enough. Clin J Am Soc Nephrol. 2008;3(1):253-260. doi: 10.2215/CJN.01430307
17. Catala-Lopez F, Tobias A, Cameron C, Moher D, Hutton B. Network meta-analysis for comparing treatment effects of multiple interventions: an introduction. Rheumatol Int. 2014;34(11):1489-1496. doi: 10.1007/s00296-014-2994-2
18. Hutton B, Salanti G, Chaimani A, Caldwell DM, Schmid C, Thorlund K, Mills E, Catala-Lopez F, Turner L, Altman DG, Moher D. The quality of reporting methods and results in network meta-analyses: an overview of reviews and suggestions for improvement. PLoS One. 2014;9(3):e92508. doi: 10.1371/journal.pone.0092508
19. Hoaglin DC, Hawkins N, Jansen JP, Scott DA, Itzler R, Cappelleri JC, Boersma C, Thompson D, Larholt KM, Diaz M, Barrett A. Conducting indirect-treatment-comparison and network-meta-analysis studies: report of the ISPOR Task Force on Indirect Treatment Comparisons Good Research Practices: part 2. Value Health. 2011;14(4):429-437. doi: 10.1016/j.jval.2011.01.011
20. Kim H, Gurrin L, Ademi Z, Liew D. Overview of methods for comparing the efficacies of drugs in the absence of head-to-head clinical trial data. Br J Clin Pharmacol. 2014;77(1):116-121. doi: 10.1111/bcp.12150
21. Fisher LD, Gent M, Buller HR. Active-control trials: how would a new agent compare with placebo? A method illustrated with clopidogrel, aspirin, and placebo. Am Heart J. 2001;141(1):26-32.
22. Pocock SJ, Gersh BJ. Do current clinical trials meet society's needs?: a critical review of recent evidence. J Am Coll Cardiol. 2014;64(15):1615-1628. doi: 10.1016/j.jacc.2014.08.008

23. Bhatnagar N, Lakshmi PV, Jeyashree K. Multiple treatment and indirect treatment comparisons: An overview of network meta-analysis. Perspect Clin Res. 2014;5(4):154-158. doi: 10.4103/2229-3485.140550
24. Bucher HC, Guyatt GH, Griffith LE, Walter SD. The results of direct and indirect treatment comparisons in meta-analysis of randomized controlled trials. J Clin Epidemiol. 1997;50(6):683-691.
25. Hasselblad V. Meta-analysis of multitreatment studies. Med Decis Making. 1998;18(1):37-43.
26. Hassan S, Ravishankar N, Nair NS. Methodological considerations in network meta-analysis. Int J Med Sci Public Health. 2015;4:588-594. doi: 10.5455/ijmsph.2015.210120151
27. Lumley T. Network meta-analysis for indirect treatment comparisons. Stat Med. 2002;21(16):2313-2324.
28. Lu G, Ades AE. Combination of direct and indirect evidence in mixed treatment comparisons. Stat Med. 2004;23(20):3105-3124.
29. Caldwell DM. An overview of conducting systematic reviews with network meta-analysis. Syst Rev. 2014;3:109. doi: 10.1186/2046-4053-3-109
30. Hutton B, Salanti G, Caldwell DM, Chaimani A, Schmid CH, Cameron C, Ioannidis JPA, Straus S. The PRISMA extension statement for reporting of systematic reviews incorporating network meta-analyses of health care interventions: checklist and explanations. Ann Intern Med. 2015;162(11):777-84. doi: 10.7326/M14-2385
31. Salanti G, Higgins JP, Ades AE, Ioannidis JP. Evaluation of networks of randomized trials. Stat Methods Med Res. 2008;17(3):279-301.
32. Veroniki AA, Vasiliadis HS, Higgins JP, Salanti G. Evaluation of inconsistency in networks of interventions. Int J Epidemiol. 2013;42(1):332-345. doi: 10.1093/ije/dys222
33. Greco T, Edefonti V, Biondi-Zoccai G, Decarli A, Gasparini M, Zangrillo A, Landoni G. A multilevel approach to network meta-analysis within a frequentist framework. Contemp Clin Trials. 2015;42:51-59. doi: 10.1016/j.cct.2015.03.005
34. Van Valkenhoef G, Dias S, Ades AE, Welton NJ. Automated generation of nodesplitting models for assessment of inconsistency in network meta-analysis. Res Synth Methods. 2016;7(1):80-93. doi: 10.1002/jrsm.1167
35. Efthimiou O, Debray TPA, Van Valkenhoef G, Trelle S, Panayidou K, Moons KGM, Reitsma JB, Shangg A, Salanti G. GetReal in network meta-analysis: a review of the methodology. Res Synth Methods. 2016;7(3):236-263. doi: 10.1002/jrsm.1195
36. Chaimani A, Higgins JP, Mavridis D, Spyridonos P, Salanti G. Graphical tools for network meta-analysis in STATA. PLoS One. 2013;8(10):e76654. doi: 10.1371/journal.pone.0076654
37. Nikolakopoulou A, Mavridis D, Salanti G. Planning future studies based on the precision of network meta-analysis results. Stat Med. 2016;35(7):978-1000. doi: 10.1002/sim.6608
38. Salanti G. Indirect and mixed-treatment comparison, network, or multiple-treatments meta-analysis: many names, many benefits, many concerns for the next generation evidence synthesis tool. Res Synth Methods. 2012;3(2):80-97. doi: 10.1002/jrsm.1037
39. Riaz IB, Khan MS, Riaz H, Goldberg RJ. Disorganized systematic reviews and meta-analyses: time to systematize the conduct and publication of these study overviews? Am J Med. 2016;129(3):339.. doi: 10.1016/j.amjmed.2015.10.009
40. Greco T, Edefonti V, Biondi-Zoccai G, Decarli A, Gasparini M, Zangrillo A, Landoni G. A multilevel approach to network meta-analysis within a frequentist framework. Contemp Clin Trials. 2015;42:51-59. doi: 10.1016/j.cct.2015.03.005
41. Madden LV, Piepho HP, Paul PA. Statistical models and methods for network meta-analysis. Phytopathology. 2016;106(8):792-806. doi: 10.1094/PHYTO-12-15-0342-RVW
42. Ohlssen D, Price KL, Xia HA, Hong H, Kerman J, Fu H, Quartey G, Heilmann CR, Ma H, Carlin BP. Guidance on the implementation and reporting of a drug safety Bayesian network meta-analysis. Pharm Stat. 2014;13(1):55-70. doi: 10.1002/pst.1592
43. Kibret T, Richer D, Beyene J. Bias in identification of the best treatment in a Bayesian network meta-analysis for binary outcome: a simulation study. Clin Epidemiol. 2014;6:451-460. doi: 10.2147/CLEP.S69660
44. Uhlmann L, Jensen K, Kieser M. Bayesian network meta-analysis for cluster randomized trials with binary outcomes. Res Synth Methods. 2016 [Epub ahead of print] doi: 10.1002/jrsm.1210
45. Mavridis D, White IR, Higgins JP, Cipriani A, Salanti G. Allowing for uncertainty due to missing continuous outcome data in pairwise and network meta-analysis. Stat Med. 2015;34(5):721-741. doi: 10.1002/sim.6365
46. Sturtz S, Bender R. Unsolved issues of mixed treatment comparison meta-analysis: network size and inconsistency. Res Synth Methods. 2012;3(4):300-311. doi: 10.1002/jrsm.1057
47. Dias S, Welton NJ, Caldwell DM, Ades AE. Checking consistency in mixed treatment comparison meta-analysis. Stat Med. 2010;29(7-8):932-944. doi: 10.1002/sim.3767
48. Sobieraj DM, Cappelleri JC, Baker WL, Phung OJ, White CM, Coleman CI. Methods used to conduct and report Bayesian mixed treatment comparisons published in the medical literature: a systematic review. BMJ Open. 2013 Jul 21;3(7):e003111. doi: 10.1136/bmjopen-2013-003111
49. Jansen JP, Crawford B, Bergman G, Stam W. Bayesian meta-analysis of multiple treatment comparisons: an introduction to mixed treatment comparisons. Value Health. 2008;11(5):956-964. doi: 10.1111/j.1524-4733.2008.00347.x
50. Jansen JP, Fleurence R, Devine B, Itzler R, Barrett A, Hawkins N, Lee K, Boersma C, Annemans L, Cappelleri JC. Interpreting indirect treatment comparisons and network meta-analysis for health-care decision making: report of the ISPOR Task Force on Indirect Treatment Comparisons Good Research Practices: part 1. Value Health. 2011;14(4):417-428. doi: 10.1016/j.jval.2011.04.002
51. Van Valkenhoef G, Lu G, Brock B, Hillege H, Ades AE, Welton NJ. Automating network meta-analysis. Res Synth Methods. 2012;3(4):285-299. doi: 10.1002/jrsm.1054
52. Greco T, Landoni G, Biondi-Zoccai G, D'Ascenzo F, Zangrillo A. A Bayesian network meta-analysis for binary outcome: how to do it. Stat Methods Med Res. 2016;25(5):1757-1773.

53. Stephenson M, Fleetwood K, Yellowlees A. Alternatives to Winbugs for network meta-analysis. Value Health. 2015;18(7):A720. doi: 10.1016/j.jval.2015.09.2730
54. Law M, Jackson D, Turner R, Rhodes K, Viechtbauer W. Two new methods to fit models for network meta-analysis with random inconsistency effects. BMC Med Res Methodol. 2016;16:87. doi: 10.1186/s12874-016-0184-5
55. Rucker G, Schwarzer G. Automated drawing of network plots in network meta-analysis. Res Synth Methods. 2016;7(1):94-107.
56. Neupane B, Richer D, Bonner AJ, Kibret T, Beyene J. Network meta-analysis using R: a review of currently available automated packages. PLoS One. 2014;9(12):e115065. doi: 10.1371/journal.pone.0115065
57. Liberati A, Altman DG, Tetzlaff J, Mulrow C, Gotzsche PC, Ioannidis JP, Clarke M, Devereaux PJ, Kleijnen J, Moher D. The PRISMA statement for reporting systematic reviews and meta-analyses of studies that evaluate healthcare interventions: explanation and elaboration. BMJ. 2009;339:b2700. doi: 10.1136/bmj.b2700
